# Mass Cytometry Study on Hepatic Fibrosis and Its Drug-Induced Recovery Using Mouse Peripheral Blood Mononuclear Cells

**DOI:** 10.3389/fimmu.2022.814030

**Published:** 2022-02-09

**Authors:** Jiwon Bae, Ji Eun Kim, Haribalan Perumalsamy, Sehee Park, Yun Kim, Dae Won Jun, Tae Hyun Yoon

**Affiliations:** ^1^Department of Chemistry, College of Natural Sciences, Hanyang University, Seoul, South Korea; ^2^Department of Internal Medicine, Hanyang University Hospital, Seoul, South Korea; ^3^Research Institute for Convergence of Basic Science, Hanyang University, Seoul, South Korea; ^4^Hanyang Medicine-Engineering-Bio Collaborative & Comprehensive Center for Drug Development, Hanyang University, Seoul, South Korea; ^5^Department of Clinical Pharmacology and Therapeutics, Hanyang University Hospital, Seoul, South Korea; ^6^Department of Medical and Digital Engineering, College of Engineering, Hanyang University, Seoul, South Korea; ^7^Institute of Next Generation Material Design, Hanyang University, Seoul, South Korea; ^8^Yoon Idea Lab. Co. Ltd, Seoul, South Korea

**Keywords:** liver fibrosis, mass cytometry, deep immune profiling, thioacetamide, PhenoGraph, FlowSOM

## Abstract

The number of patients with liver diseases has increased significantly with the progress of global industrialization. Hepatic fibrosis, one of the most common liver diseases diagnosed in many developed countries, occurs in response to chronic liver injury and is primarily driven by the development of inflammation. Earlier immunological studies have been focused on the importance of the innate immune response in the pathophysiology of steatohepatitis and fibrosis, but recently, it has also been reported that adaptive immunity, particularly B cells, plays an essential role in hepatic inflammation and fibrosis. However, despite recent data showing the importance of adaptive immunity, relatively little is known about the role of B cells in the pathogenesis of steatohepatitis fibrosis. In this study, a single-cell-based, high-dimensional mass cytometric investigation of the peripheral blood mononuclear cells collected from mice belonging to three groups [normal chow (NC), thioacetamide (TAA), and 11beta-HSD inhibitor drug] was conducted to further understand the pathogenesis of liver fibrosis through reliable noninvasive biomarkers. Firstly, major immune cell types and their population changes were qualitatively analyzed using UMAP dimensionality reduction and two-dimensional visualization technique combined with a conventional manual gating strategy. The population of B cells displayed a twofold increase in the TAA group compared to that in the NC group, which was recovered slightly after treatment with the 11beta-HSD inhibitor drug. In contrast, the populations of NK cells, effector CD4^+^ T cells, and memory CD8^+^ T cells were significantly reduced in the TAA group compared with those in the NC group. Further identification and quantification of the major immune cell types and their subsets were conducted based on automated clustering approaches [PhenoGraph (PG) and FlowSOM]. The B-cell subset corresponding to PhenoGraph cluster PG#2 (CD62L^high^CD44^high^Ly6c^high^ B cells) and PG#3 (CD62L^high^CD44^high^Ly6c^low^ B cell) appears to play a major role in both the development of hepatic fibrosis and recovery *via* treatment, whereas PG#1 (CD62L^low^CD44^high^Ly6c^low^ B cell) seems to play a dominant role in the development of hepatic fibrosis. These findings provide insights into the roles of cellular subsets of B cells during the progression of, and recovery from, hepatic fibrosis.

## Introduction

Among the many abnormalities in the liver, hepatic fibrosis is one of the most common liver diseases in many developed countries (1–4). The disease occurs in response to chronic liver injury and is mostly driven by the development of inflammation. The primary causes of hepatic fibrosis include alcohol abuse, non-alcoholic steatohepatitis (NASH), and hepatitis C virus (HCV) infection. At the molecular level, hepatic fibrosis is a dynamic process caused by an imbalance in the synthesis and degradation of the extracellular matrix (ECM) and is often characterized by the excessive accumulation of ECM proteins in the liver (5). Recent immunological studies on hepatic fibrosis have revealed different roles of both adaptive and innate immune systems in the regulation of hepatic fibrosis. It is well known that individuals with hepatic fibrosis have a high risk of developing cirrhosis (6, 7), where the excessive accumulation of ECM proteins distorts the hepatic architecture *via* the formation of fibrous scars and subsequent formation of nodules by the regenerating hepatocytes.

Many studies involving the development of improved diagnostic tools and therapeutic treatments for hepatic fibrosis and cirrhosis are currently in progress. Invasive liver biopsy is considered the gold standard approach for the diagnosis of hepatic fibrosis and cirrhosis, whereas imaging methods (e.g., ultrasonography) are generally used to detect moderate to severe fibrosis (8). However, liver biopsy is an invasive process involving serious pain and major complications in patients, and it may be accompanied by sampling error, as the biopsy samples represent only a small region of the liver, rather than representing the state of the entire liver. Therefore, there is an urgent need to develop non-invasive and accurate methods for the diagnosis of these liver diseases. Although non-invasive and inexpensive, ultrasonography is highly dependent on operators, and challenges in reliably differentiating hepatic steatosis from fibrosis using ultrasonography have been reported (9). Despite many studies reporting the potential reversibility of hepatic fibrosis and cirrhosis (2), apart from liver transplantation, there is no clear treatment for these diseases. This is primarily due to the gaps in the understanding of the pathogenesis and etiology of hepatic fibrosis. Additionally, although significant advancements have been made in animal studies to prevent the progression of fibrosis (10), the efficacy of most therapeutic treatments for humans is yet to be confirmed, owing to the lack of non-invasive diagnostic methods that accurately assess changes in liver fibrosis status. Therefore, there is an urgent need to develop a non-invasive, accurate, and reliable method for the diagnosis of hepatic fibrosis, based on an improved understanding of its pathogenesis, as well as to develop reliable noninvasive biomarkers of liver fibrosis.

Recent immunological studies on hepatic fibrosis have revealed the different roles of various immune cells in the regulation of hepatic fibrosis. For instance, the innate immune system participates in the regulation of hepatic fibrosis *via* Kupffer cells (KCs), natural killer (NK) cells, and dendritic cells (DCs). KCs, hepatic macrophages derived from circulating monocytes, constitute 15% of the total liver cell population, and the phenotypes and functions of these hepatic macrophages are critical determinants in the progression of hepatic fibrosis. Inflammatory stimuli can differentiate monocytes into M1 or M2 macrophages. Activation of M1 macrophages results in the release of pro-inflammatory cytokines, propagation of inflammation, and eventually the development of fibrosis, whereas M2 macrophages release anti-inflammatory cytokines that promote cell proliferation and reduce apoptosis. NK cells and natural killer T (NKT) cells, which are known to provide the foremost line of defense against invading antigens, constitute a large proportion of liver-resident lymphocytes and protect the liver against fibrosis by inducing hepatic stellate cell (HSC) apoptosis and production of antifibrotic mediators. DCs represent ~25% of leukocytes in fibrotic hepatic tissue and are known to play central roles in the modulation of liver immunity. Connolly et al. (10), based on their study on a mouse model of liver fibrosis, reported a fivefold increase in the hepatic DC population with enhanced capability to stimulate NK cells, T cells, and HSCs (11). Removal of DCs has also been reported to deplete inflammatory mediators in the fibrotic liver. The adaptive immune system is also considered to play an important role in the pathogenesis of hepatic fibrosis. In particular, the role of B cells is gaining more attention, with an increasing number of studies investigating B-cell function. An increase in B-cell population promoted fibrosis in animal models of acute liver injury (12, 13). In a study on CCl_4_-induced fibrosis, Novobrantseva et al. (11) reported reduced collagen deposition in B-cell-deficient mice. Accumulation of B cells in NASH livers has also been reported with inflammation and fibrosis, wherein B cells secrete elevated levels of pro-inflammatory cytokines with antigen-presentation ability (14, 15). Previous studies (16, 17) have reported that the ratio of Th1/Th2 lymphocytes might determine the role of T cells in favor of or against hepatic fibrosis, as Th1 cells secrete antifibrotic cytokines (e.g., IFN-γ) and Th2 cells secrete profibrotic cytokines (e.g., IL-4).

Among the components of the human immune system, peripheral blood mononuclear cells (PBMCs), comprising a diverse mixture of highly specialized immune cells, are known to play a key role in fighting infection. PBMCs are considered as an important tool for researchers and clinicians working on human health and disease, as they can be used to test immune responses of the body to various external stimuli and improve our understanding of various diseases, as well as help us to develop new therapeutic treatments and assess the efficacy of newly developed drugs. In the development of the diagnosis or prognosis of hepatic fibrosis, immune profiling of PBMCs are considered as an alternative method with a non-invasive, less complicated more accurate assessing capabilities (18–21). For a better understanding of different stages of liver steatosis and fibrosis (e.g., NAFLD, NASH, cirrhosis, and hepatic carcinoma), deep immune-profiling of cellular composite (e.g., non T cells such as monocytes, dendritic cells, NK cells, B cells, and T cells including CD4+, CD8+, regulatory, and cytotoxic killer T cells) cells from PBMCs of patients may provide the significant and unconventional way. The proinflammatory cytokine-mediated responses such as IL-2, IL-5, IL-6, and IFN execute activation of monocytes and lymphocytes, which can abundantly secrete inflammatory cytokines and release them into blood serum to initiate liver fibrosis through hyper-inflammatory responses, immune cellular differentiation, coagulation, and another liver steatosis.

In these immunological studies of hepatic fibrosis, fluorescence-based flow cytometry, which differentiates each immune cell based on the expression of surface proteins, is a widely used method for monitoring the immune response. However, fluorescence-based flow cytometry has drawbacks, with respect to providing sufficient cellular information on immune systems comprised of highly heterogeneous cells with multiple phenotypes and expression of various proteins. Although many studies have been conducted on various liver diseases using fluorescence-based flow cytometry or fluorescence microscopy (22–27), it is difficult to fully understand the complex and interconnected changes in immune cell populations related to hepatic fibrosis using fluorescence-based flow cytometry, owing to the small number of dimensions limited by the spectral overlaps between individual fluorescence channels. Mass cytometry (CyTOF) is a novel single-cell-based, high-dimensional cytometry technique that overcomes the limitations of conventional fluorescence-based flow cytometry (28–31). This technology detects antibodies tagged with lanthanide metal isotope ions (with atomic weights ranging from 75 to 209) to examine ~50 markers (including surface or intracellular proteins) intended for detection simultaneously at a single-cell resolution (30). As such, high-dimensional technology enables a more comprehensive understanding of immune responses, and several clinical studies are currently underway for exploring immune responses against diseases associated with PBMC or bone marrow (31–33). The ability of high-dimensional techniques to detect multiple metal-tagged biomarkers with minimal overlap and cellular background interference provide potential applications in several areas of biomedical research, such as deep phenotyping of heterogeneous cells, mapping of cell differentiation, and disease progression to generate high-resolution profiles of the cell cycle, studying differential cytokine expression, and studying signaling response.

However, the visualization and interpretation of high-dimensional data produced by mass cytometry are extremely challenging. Therefore, it is necessary to adapt advanced data analysis algorithms for dimensionality reduction or automated clustering. For instance, dimensionality reduction algorithms, such as t-distributed stochastic neighbor embedding (t-SNE) or uniform manifold approximation and projection (UMAP) in the representation of high-dimensional mass cytometry data in easy-to-understand two-dimensional (2D) plots, and automated clustering algorithms, such as PhenoGraph and FlowSOM, can be used to distinguish cellular clusters based on their attribute characteristics. New insights into the different roles of various immune cells in the regulation of hepatic fibrosis can be elucidated *via* the application of these advanced data analysis tools on high-dimensional mass cytometric data, which are otherwise difficult to grasp for human investigators. Therefore, mass cytometry, combined with high-dimensional data analysis algorithms, provides insights into complex immune systems and enables a more comprehensive understanding of their heterogeneity.

In this study, single-cell-based, high-dimensional mass cytometric measurements were performed on the PBMCs collected from mice in the following three groups: normal chow (NC), thioacetamide (TAA)-induced liver fibrosis, and 11beta-HSD inhibitor drug (Drug)-treated. A qualitative exploration of the major immune cell distribution changes was performed using dimensionality reduction and 2D visualization techniques such as UMAP and minimum spanning tree (MST), combined with a conventional manual gating strategy. Further identification of major immune cell types and their subsets was also conducted based on automated clustering approaches including PhenoGraph and FlowSOM, followed by comparison of their cellular abundances among the three groups, to provide useful insight into liver fibrosis-related immune cell subsets and to test the efficacy of drugs intended for liver fibrosis treatment.

## Materials and Methods

### Animals

C57BL/6N mice (6 weeks old, male) were obtained from Orient Bio (Seongnam-si, Gyeonggi-do, South Korea). The mice were maintained in a temperature-controlled (23°C ± 2°C) and specific-pathogen-free room under a 12-h light/dark cycle. The objectives and the experimental procedures were approved based on ethical guidelines provided by the Institutional Animal Care and Use Committee of Hanyang University. The ethical approval number is 2019-0176A (HY-IACUC-21-0001).

### Drug

J2H-1702, the drug used in this study, was kindly provided by J2H Biotech (Suwon, South Korea). J2H-1702 is known to function as an inhibitor of 11beta-HSD for recovery from liver fibrosis and is currently in phase 1 clinical trial (CRIS Registration Number: KCT0005295) for liver fibrosis treatment. 11beta-HSD is a cortisone reductase that converts inactive cortisone to active cortisol and promotes fat deposition and formation in the liver when it is overexpressed. According to previous studies (34–37), the inhibition or deficiency of 11beta-HSD is effective to induce liver fibrosis and it plays an important role for treating liver fibrosis if we could use it as a drug.

### Experimental Design

At 6 weeks of age, the animals were divided into the following three groups (at Hanyang University Animal Laboratory): normal chow mice (NC, *n* = 7); thioacetamide (TAA)-induced liver fibrosis mice (TAA, *n* = 8); and drug-treated mice (Drug, *n* = 8). These three groups were annotated as NC (normal chow, control sample with normal feed), TAA (fed a thioacetamide diet that induced liver fibrosis, disease-induced sample), and Drug (fed a TAA diet and treated with a high dose of 11beta-HSD inhibitor, drug-treated sample). The NC and TAA groups were fed normal and TAA diets for 19 weeks, respectively, whereas the Drug group was fed with a TAA diet for 10 weeks and then a TAA diet combined with a high dose (10 mg/kg of mouse body mass) of 11beta-HSD inhibitor drug for another 9 weeks. In the TAA-induced liver fibrosis mouse model, it was observed that inflammation and ballooning scores through H&E staining were significantly increased [normal control vs. TAA: 1 vs. 3.53 ± 0.36 (inflammation); 1 vs. 3.84 ± 0.27 (ballooning)]. Fibrosis area was significantly increased through Sirius red staining (normal control vs. TAA: 0.46 ± 0.22 vs. 6.13 ± 1.05). In addition, liver injury markers including ALT and AST were also significantly increased in the TAA model [normal control vs. TAA: 24.75 ± 13.58 vs. 148.7 ± 89.7 (ALT); 73.73 ± 27.09 vs. 250.8 ± 105.8 (AST)]. In the meantime, hepatic gene expression of both inflammatory and fibrosis markers were significantly increased such as TNF-a, MCP1, a-SMA, Col1a1, TIMP-1, and FN-1. To provide the same environmental and stress conditions, a mixture of 1% Tween80 and 0.5% methyl cellulose (MC) in tertiary distilled water was orally administered to the NC and TAA groups, instead of 11beta-HSD inhibitor. The TAA was dissolved in water at 0.03% and the 11beta-HSD inhibitor was dissolved in 1%Tween 80 and 0.5%MC at 10 mg/kg and administrated daily as previously described (37, 38).

### Isolation of Mouse PBMCs From Whole Blood

Mouse whole blood was drawn from the heart into EDTA-treated tubes (BD Vacutainer^®^, USA), from which mPBMCs were isolated *via* density gradient centrifugation using Ficoll-Paque PLUS (GE Healthcare Bio-Sciences, Sweden). Briefly, blood was diluted in a 1:1 ratio with phosphate-buffered saline (PBS) (Welgene, Korea), and the diluted blood was overlaid on the Ficoll reagent in 15-ml centrifuge tubes. These tubes were centrifuged at 400 × *g* at 27°C for 40 min on a centrifuge with a swing-bucket rotor (Labogene, Korea). The mononuclear cell layer was then collected and transferred to a new tube, washed in PBS, and centrifuged at 350 × *g* at room temperature for 5 min. The supernatant was discarded, and the cells were resuspended in RPMI complete medium, consisting of RPMI-1640 medium (Lonza™ BioWhittaker™, USA) supplemented with 10% fetal bovine serum (Gibco, USA), and 1% penicillin/streptomycin (Gibco, USA), for subsequent treatments.

### Staining of mPBMCs

Cells were stained with lanthanide metal-tagged surface antibodies (**Table 1**) using the Maxpar Cytoplasmic/Secreted Antigen Staining with Fresh Fix kit (Fluidigm, USA), as per the manufacturer’s instructions. Briefly, the cells were washed with PBS and stained with cisplatin to assess viability. Next, mPBMCs were stained with the 19 surface markers listed in **Table 1**. After staining with these surface markers, the cells were fixed with 1.6% formaldehyde and stained with Cell-ID Intercalator-Ir. Prior to data acquisition, the cells were washed and suspended at 1 × 10^6^ cells/ml in Cell Acquisition Solution (Fluidigm, USA). Calibration beads were added at a 1:10 ratio by volume for normalization. Cells were then filtered into strainer-capped tubes, and samples were analyzed using the Helios mass cytometry platform (Fluidigm, USA).

**Table 1:** List of metal-tagged antibodies of surface markers.

Target	Cell	Metal	Target	Cell	Metal
Ly-6G/C/Gr-1	Granulocytes	141Pr	TER-119	Red blood cell	154Sm
CD11c	DC	142Nd	CD62L	Activated T cell	160Gd
CD69	Activated T,B,NK	145Nd	CD8a	T cell	168Er
CD45	Leukocytes	147Sm	TCR*β*	*αβ* T cell	143Nd
CD11b/Mac-1	Macrophage	148Nd	NK1.1	NK cell	170Er
CD19	B cell	149Sm	CD44	Activated T cell	171Yb
CD25	T reg	151Eu	CD4	T cell	172Yb
CD3e	T cell	152Sm	B220	B cell	176Yb
F4/80	Macrophage	159Tb	Ly6c	Macrophage	162Dy
CD206	Macrophage	169Tm			

### Mass Cytometer Setup, Calibration, and Data Acquisition

A Helios mass cytometer (Fluidigm, USA) was used for data acquisition. The instrument was tuned by optimizing the nebulizer, makeup gas, current, and detector voltage, according to the manufacturer’s guidelines. To acquire data of mPBMC samples, the instrument was set to “event mode”, while injection speed was kept at 5 × 10^-7^ L/s and push length was set at 13 µs by default.

### Mass Cytometry Data Analysis

FlowJo (FlowJo, LLC, USA) and Cytobank (Cytobank, Inc., USA) were used for data analysis and visualization. Inverse hyperbolic sine (arcsinh) transformation was applied to the raw data, and preprocessing steps were performed to remove signals from beads, doublets, and dead cells. The major immune cell types and their subsets were then manually gated based on the gating strategy presented in **Figure S1**. The UMAP method was used to reduce dimensionality and visualize data in single-cell resolution. In addition to the manual gating approach, automated clustering methods, such as the PhenoGraph and FlowSOM methods, were adopted in this study. PhenoGraph clustering was performed using a plugin of FlowJo S/W with a *k* value of 30, while the FlowSOM clustering method was performed using Cytobank S/W with a metacluster number of 20. Statistical significance was assessed using the Mann–Whitney *U* test. Differences with *p* < 0.05 were considered statistically significant, and 0.01 < *p* < 0.05, 0.001 < *p* < 0.01, and *p* < 0.001 have been annotated as *, **, and ***, respectively.

## Results

### Mass Cytometry Workflow

**Figure 1:**
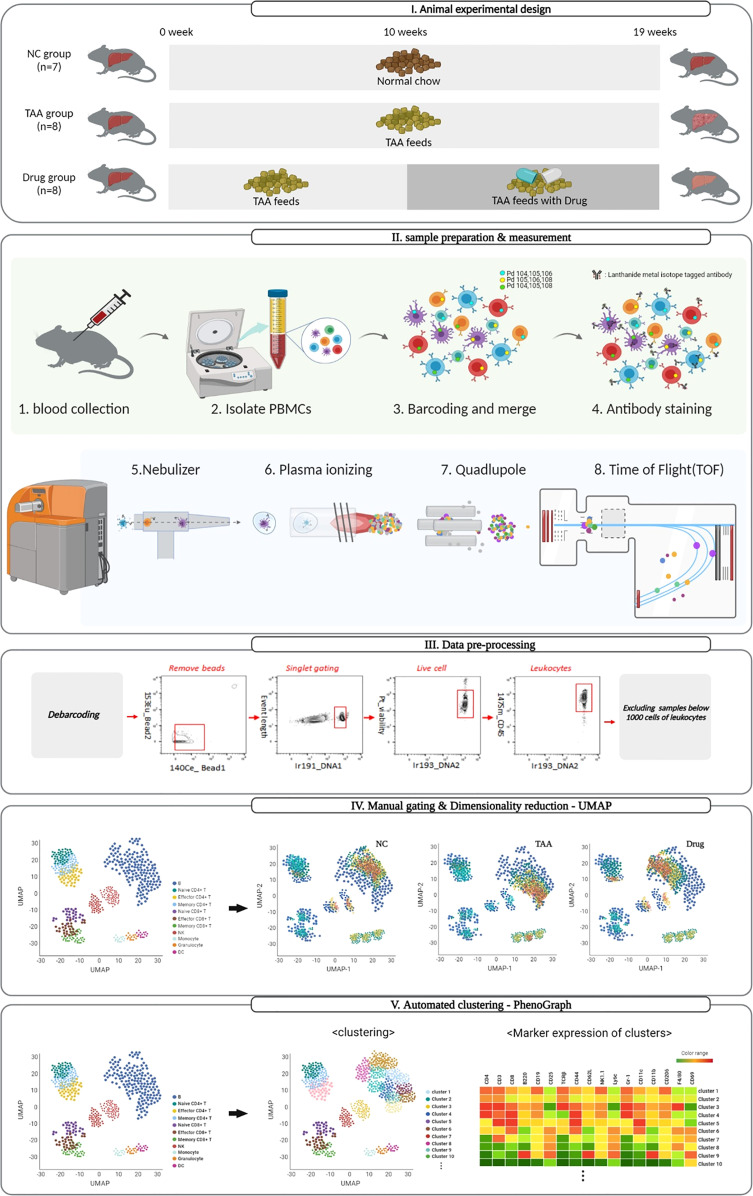
Schematic representation of the mass cytometry workflow and dimensionality visualization performed by UMAP, PhenoGraph, and FlowSOM clustering to visualize data in single-cell resolution in mouse PBMC cells.

A schematic overview of the mass cytometry workflow used in this study is shown in **Figure 1**. The mPBMCs isolated from blood samples of 23 mice, which consisted of 7, 8, and 8 mice from the NC, TAA, and Drug groups, respectively, were stained and analyzed using single-cell-based mass cytometry. The high-dimensional mass cytometry data were then analyzed as follows. First, preprocessing steps were performed to clean up the mass cytometry data by removing signals from beads, non-singlets, and dead cells. Subsequently, based on the manual gating strategy shown in **Figure S1**, six major cell phenotypes (i.e., B cells, CD4^+^T cells, CD8^+^T cells, dendritic cells, monocytes, and NK cells) with a few subsets (i.e., Naïve/Effector/Memory CD4^+^T and CD8^+^T cells) were manually assigned, and dimensionality reduction and 2D visualization of the mass cytometry data were performed to qualitatively explore variations in immune cell populations among the NC, TAA, and Drug groups. Following this, the identification of immune cell types and their subsets was conducted to obtain cellular abundance profiles of the immune cells, based on the conventional manual gating strategy, as well as using automated clustering algorithms PhenoGraph and FlowSOM. Finally, to identify liver fibrosis-related immune cell types and their subsets and to assess the immunomodulatory effects of 11beta-HSD inhibitor, the cellular abundances were quantitatively compared among the three groups.

### Qualitative Comparison of the NC, TAA, and Drug Group Mice Using UMAP and FlowSOM MST Visualization

**Figure 2:**
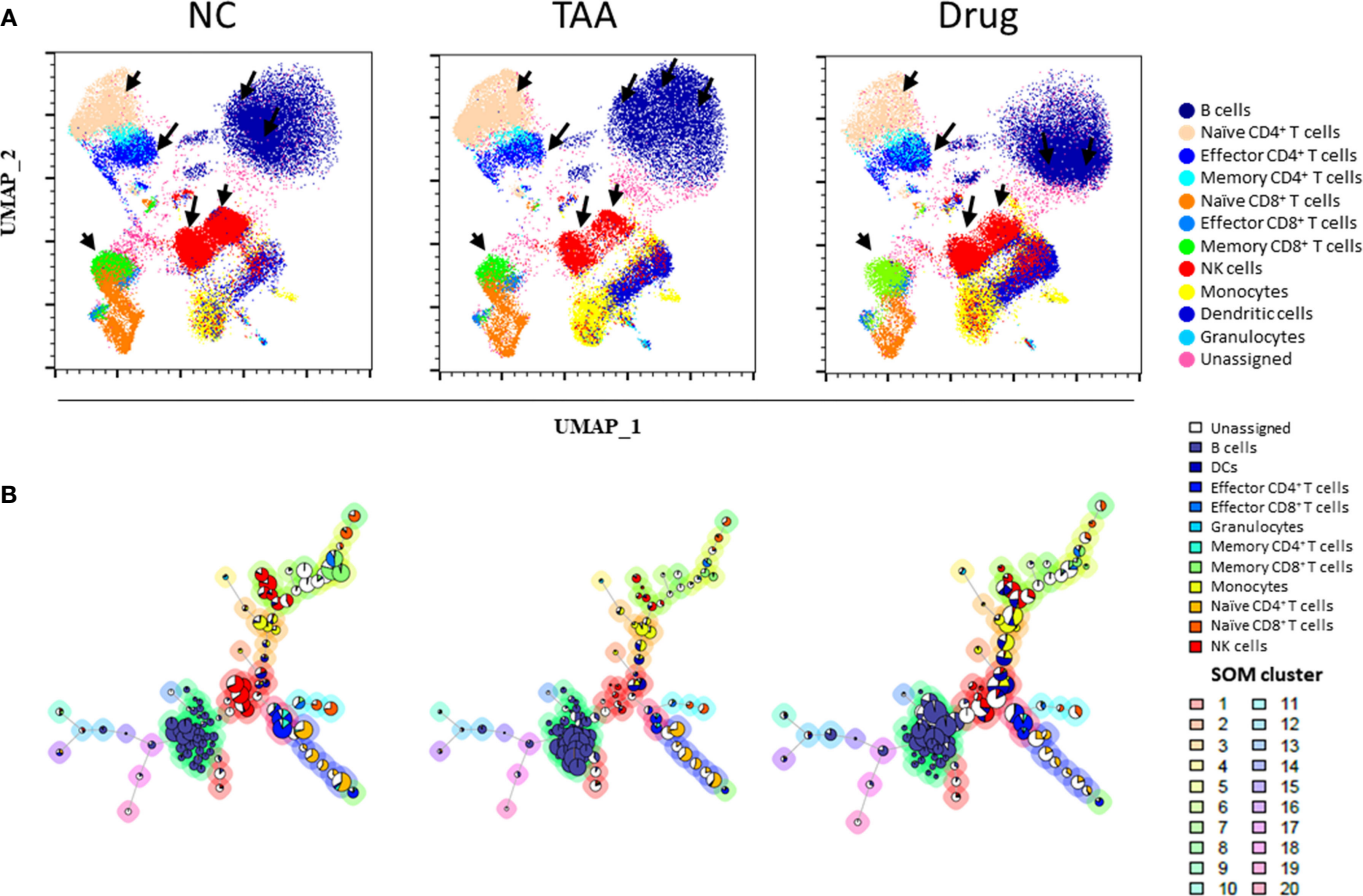
Uniform manifold approximation and projection (UMAP) visualization of **(A)** the immune leukocytes population density between NC, TAA, and Drug groups. The arrows indicate the UMAP manually gated clusters with significant differences between the NC, TAA, and Drug groups. **(B)** Population pie graph of the NC, TAA, and Drug groups on FlowSOM minimum spanning trees (MSTs). The pie chart representation of population abundance aggregated with NC, TAA, and Drug groups.

Owing to the UMAP’s advantage of preserving inter-cluster (or global) information, cell types with similar characteristics were located close to each other, and the characteristics of the unassigned population from the manual-gating process could be inferred from their neighboring cell types. As shown in **Figure 2A**, the manually gated immune cell types overlaid on UMAP visualization formed five distinct regions, namely, B cells in the top-right region; monocytes, DCs, and granulocytes in the bottom right region; NK cells at the central region; CD4^+^ T cells at the top left region; and CD8^+^ T cells at the bottom left region of UMAP, while unassigned populations from manual gating were observed between the islands of manually gated cell types [e.g., gray dots observed between the islands of B cells (dark blue) and NK cells (red)]. All the T cells were located on the left side of the plot, whereas the CD4^+^ and CD8^+^ T cells were separated into the top and bottom regions of the left side. Moreover, the subsets of CD4^+^ and CD8^+^ T cells, such as effector, memory, and naïve cells, were located more closely than the rest of the clusters. Additionally, preservation of the inter-cluster information in UMAP was also observed where dendritic cells and monocytes, which are known to share many similarities with each other, were located.

Additionally, cell population density distributions within the same cell types showed significant variations among the groups. Specifically, B cells, NK cells, and CD4^+^ T cells had a distinct distribution among the NC, TAA, and Drug groups (**Figure 2A**). Notable differences were found in B-cell islands between three groups; in particular, the NC group displayed highly concentrated density population distribution on the left region of the B-cell islands, while the TAA group showed a highly concentrated population in the upper region and the drug-treated group showed a highly concentrated population in the bottom regions of the B-cell islands. Similarly, the subset naïve CD4^+^T cells showed an increase in the population density of the TAA group while the NC group has shown a slight reduction in population density. In contrast, the Drug group showed an intense population density reduction compared with the TAA group. In the case of the effector CD4^+^ T cells of the NC group, we observed a significant distribution at the bottom of the island, whereas the TAA group displayed much smaller distribution. However, the Drug groups denoted recovery of density distribution of effector CD4^+^ T cells (**Figure 2A**). NK cells also displayed a dramatic reduction in cell density in the TAA group compared to the NC group, although it partially returned in the Drug group.

As shown in **Figure 2B**, manually gated cell populations were also overlaid on an MST produced using FlowSOM. Similar to the observations in **Figure 2A**, distinct shifts and population changes within the same cell types, particularly for B cells and NK cells, were observed among the groups. In the case of B cells, remarkable increases in the sizes of the pie charts located in the lower part of the B-cell branches were observed in the TAA group, which were then shifted to the upper part of the B-cell branches for the Drug group. In contrast, NK cell nodes of the TAA group displayed a significant decrease in the size of the pie charts compared to the NC group, whereas the reduced NK cell population appeared to have recovered in the Drug group (**Figure S6**). To further investigate variations in the immune cell subset populations among the groups, we adapted automated clustering algorithms, namely, PhenoGraph and FlowSOM, in addition to a conventional manual gating strategy, and quantified the abundance of immune cells and their subsets, to analyze immune cell subsets related to hepatic fibrosis and to assess the immunomodulatory effects of the drug used in this study.

### Comparison of Manually Gated Cellular Abundances Among the Three Groups

**Figure 3:**
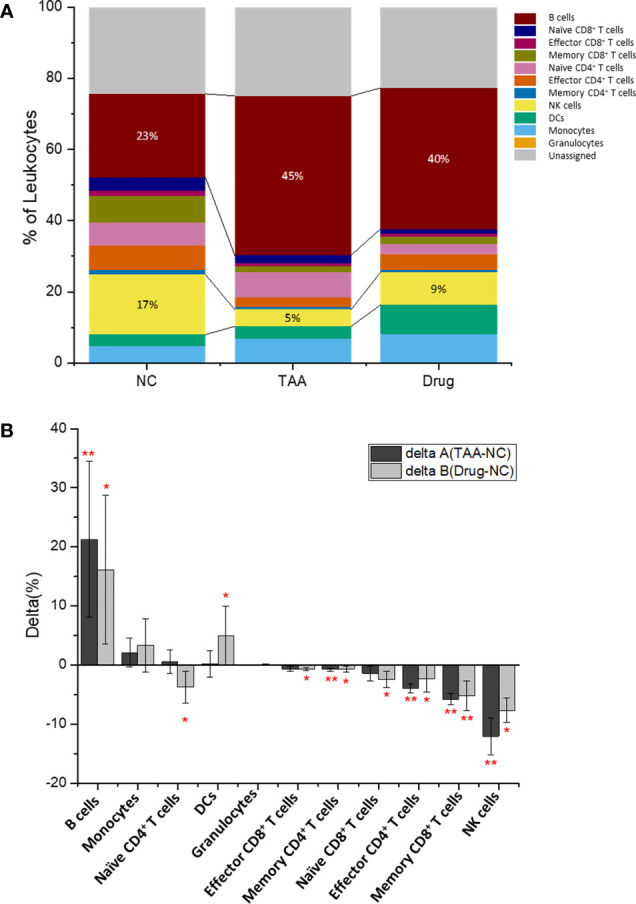
Immune leukocytes population. **(A)** Population abundance (% of leukocytes) of the manually gated cell types of NC, TAA, and Drug groups. **(B)** Delta (%) calculation of delta A (TAA-NC) and delta B (Drug-NCC) by comparing and values. The statistical significant differences considered 0.01 < *p* < 0.05, and 0.001 < *p* have been annotated as * and ** respectively.

The quantified cellular abundances of the manually gated cell types are summarized in **Figure 3**, **Figure S3**, and **Table S1**. Several major immune cell types were found to be differentially abundant among the three groups. For instance, the abundance of B-cell population showed a twofold increase in the TAA group [44.7%, delta(TAA-NC) = +21.2%] compared to that in the NC group (23.5%), although the increased value was slightly reduced after treatment with 11beta-HSD inhibitor [Drug group: 39.6%, delta(Drug-NC) = +16.1%]. In contrast, the population of NK cells was dramatically reduced in the TAA group [4.76%, delta(TAA-NC) = −12.0%] compared to that in the NC group (16.8%), although compared to this decreased value, a slight increase was noted in the Drug group [9.14%, delta(Drug-NC) = −7.69%].

The populations of effector CD4^+^ and memory CD8^+^ T cells in the TAA group also showed significantly reduced abundances (2.69% and 1.59% for the effector CD4^+^ and memory CD8^+^ T cells, respectively) compared to those in the NC group (6.69% and 7.38%, respectively) with delta(TAA-NC) values of −4.0% and −5.8%, respectively. Similar to the aforementioned results on B cells and NK cells, the 11beta-HSD inhibitor-treated group exhibited slight recovery in abundances [4.33% abundance with delta(Drug-NC) = −2.36% and 2.16% abundance with delta(Drug-NC) = −5.22%, for effector CD4^+^ and memory CD8^+^ T cells, respectively]. Correspondingly, the naïve CD4+ T cells showed dramatic population increment in the TAA group, compared with the Drug group while the NC group showed slight reduction. The reduced populations of effector CD4^+^ T cells and NK cells might impair the process of elimination of activated HSC cells or retained hepatocytes, potentially leading to progression of hepatic fibrosis to cirrhosis or hepatocellular carcinoma.

### Comparison of the Cellular Abundances of PhenoGraph and FlowSOM Clusters Among the Three Groups

**Figure 4:**
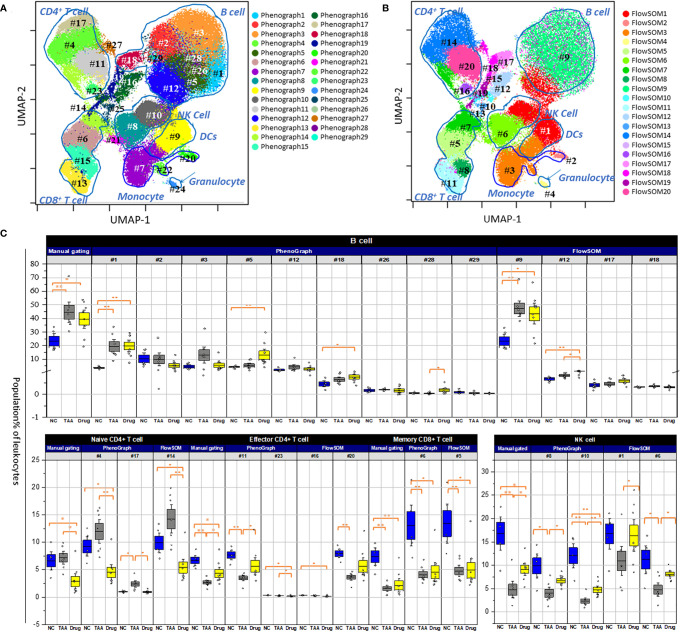
Population abundance of manual gating. **(A)** PhenoGraph clusters analysis of manual gated leukocytes population. **(B)** FlowSOM clusters analysis of manual gated leukocytes population. Both overlaid on uniform manifold approximation and projection (UMAP). The numbering with # denoted individual population clusters. **(C)** Comparison of manually gated cell types for NC, TAA, and Drug groups represented in boxplots graph. The statistical significant differences considered 0.01 < *p* and < 0.05, 0.001 < *p* have been annotated as * and ** respectively.

To further investigate the differential abundance of cellular subsets among the three groups, automated clustering algorithms (PhenoGraph and FlowSOM) were applied to identify clusters of cellular subsets and quantify their abundances. As shown in **Figure 4A** and **Table 2**, twenty-nine clusters were identified using PhenoGraph (32, 38, 39, 40) and then assigned to both their corresponding cell types, *via* manual gating, and their associated surface marker expression. Nine clusters were assigned as subsets of B cells (PG#1, PG#2, PG#3, PG#5, PG#12, PG#18, PG#26, PG#28, and PG#29), five clusters as subsets of CD4^+^ T cells [two naïve (PG#4 and PG#17), two effector (PG#11 and PG#23), and one memory (PG#27)], three clusters as subsets of CD8^+^ T cells [one naïve (PG#13), one effector (PG#15), and one memory (PG#6)], three clusters as subsets of monocytes (PG#7, PG#20, and PG#22), two clusters as subsets of NK cells (PG#8 and PG#10), one cluster as a subset of DCs (PG#9), and one cluster as a subset of granulocytes (PG#24) (**Figure S4** and **Table S2**). Among the manually gated cell types with significant variations among the three groups (as discussed in the previous section), memory CD8^+^ T cells were assigned to a single cluster (PG#6) and effector CD4^+^ T cells were found to have one cluster (PG#11) with a dominant population, while B cells were composed of multiple subsets with significant contributions. The results of FlowSOM clustering are also presented in **Figure 4B**, wherein 20 identified metaclusters have been overlaid on UMAP visualization. These FlowSOM clusters were then assigned to the corresponding manually gated cell types and surface marker expression, as shown in **Figure S5** and **Table 3**. Four metaclusters were assigned as subsets of B cells (FS#9, FS#12, FS#17, and FS#18), four metaclusters as subsets of CD4^+^ T cells (FS#7, FS#14, FS#16, and FS#20), three metaclusters as subsets of the CD8^+^ T cells (FS#5, FS#8, and FS#11), two metaclusters as subsets of the monocytes (FS#2 and FS#3), two clusters as subsets of NK cells (FS#1 and FS#6), one cluster as a subset of DCs (FS#1), and one cluster as a subset of granulocytes (FS#4) (**Figure S2** and **Table S3**).

**Table 2:** Comparison of PhenoGraph clusters with manually gated cell phenotypes and associated marker expression.

Cell type	PhenoGraph cluster	Marker expression
Naïve CD4^+^ T cell	PG#4	TCRβ^mid^CD3^high^CD4^high^CD62L^high^CD44^mid^Ly6c^high^CD206^high^
	PG#17	TCRβ^high^CD3^high^CD4^high^CD62L^high^CD44^low^Ly6c^mid^CD206^high^
Effector CD4^+^ T cell	PG#11	TCRβ^mid^CD3^high^CD4^high^CD62L^low^CD44^high^Ly6c^mid^CD69^low^
	PG#23	TCRβ^high^CD3^high^CD4^high^CD62L^mid^CD44^high^Ly6c^high^CD69^high^
Memory CD4^+^ T cell	PG#27	TCRβ^high^CD3^high^CD4^high^CD62L^mid^CD44^high^Ly6c^low^
Naïve CD8^+^ T cell	PG#13	TCRβ^mid^CD3^high^CD8^high^CD62L^high^CD44^high^Ly6c^mid^CD206^high^
Effector CD8^+^ T cell	PG#15	TCRβ^mid^CD3^high^CD8^high^CD62L^high^CD44^high^Ly6c^high^CD206^high^
Memory CD8^+^ T cell	PG#6	TCRβ^low^CD3^high^CD8^high^CD62L^high^CD44^high^Ly6c^high^CD206^mid^
B cell	PG#1	B220^high^CD19^high^CD62L^low^ CD44^high^Ly6c^low^
	PG#2	B220^high^CD19^high^CD62L^high^CD44^high^Ly6c^high^
	PG#3	B220^high^CD19^high^CD62L^high^CD44^high^Ly6c^low^
	PG#5	B220^high^CD19^high^CD62L^low^CD44^high^ Ly6c^high^
	PG#12	B220^mid^CD19^high^CD62L^low^CD44^high^Ly6c^mid^
	PG#18	B220^high^CD19^high^CD62L^high^CD44^high^ Ly6c^high^CD11b^low^CD11c^high^F4/80^high^
	PG#26	B220^high^CD19^high^CD62L^high^ CD44^high^Ly6c^mid^F4/80^high^
	PG#28	B220^high^CD19^high^NK1.1^high^CD62L^mid^CD44^high^Ly6c^mid^
	PG#29	B220^high^CD19^high^CD62L^mid^Gr-1Ly6c^mid^
NK cell	PG#8	NK1.1^high^CD11c^low^CD11b^mid^Ly6c^high^
	PG#10	NK1.1^high^CD11c^mid^CD11b^low^Ly6c^low^
Monocyte	PG#7	CD11b^high^CD11c^low^Gr-1^low^Ly6c^high^CD44^high^
	PG#20	CD11b^high^CD11c^low^Ly6c^high^F4/80^high^CD206^low^
	PG#22	CD11b^high^CD11c^low^Ly6c^high^F4/80^high^CD206^low^
Dendritic cell	PG#9	CD11b^high^CD11c^high^Ly6c^high^CD44^high^
Granulocyte	PG#24	CD11b^high^CD11c^low^Ly6c^high^Gr-1^high^F4/80^low^CD206^low^
Unassigned	PG#14	TCRβ^low^CD3^high^CD4^high^CD62L^high^CD44^high^Ly6c^high^CD206^high^
	PG#16	B220^high^CD19^high^CD62L^mid^CD44^high^Ly6c^high^CD69^high^
	PG#19	TCRβ^high^CD3^high^CD8^high^CD62L^high^CD44^high^Ly6c^high^
	PG#21	CD11c^high^CD11b^low^Ly6c^high^F4/80^low^CD206^low^
	PG#25	B220^high^CD19^high^NK1.1^high^CD62L^high^ CD44^high^Ly6c^high^Gr-1^mid^CD206^high^CD25^high^

**Table 3:** Comparison of FlowSOM clusters with manually gated cell phenotypes and associated marker expression.

Cell type	FlowSOM cluster	Marker expression
Naïve CD4^+^ T cell	FS14	TCRβ^high^CD3^high^CD4^high^CD62L^mid^CD44^mid^Ly6c^mid^CD206^high^
Effector CD4^+^ T cell	FS16	TCRβ^high^CD3^high^CD4^high^CD62L^mid^CD44^high^Ly6c^mid^CD69^high^
	FS20	TCRβ^high^CD3^high^CD4^high^CD62L^low^CD44^high^Ly6c^low^
Memory CD4^+^ T cell	FS7	TCRβ^high^CD3^high^CD4^high^CD8^mid^CD62L^high^CD44^high^Ly6c^high^CD206^high^
Naïve CD8^+^ T cell	FlS11	TCRβ^low^CD3^high^CD8^high^CD62L^low^CD44^mid^Ly6c^low^CD69^high^CD206^high^
Effector CD8^+^ T cell	FS8	TCRβ^mid^CD3^high^CD8^high^CD62L^high^CD44^low^Ly6c^low^Gr-1^high^CD206^mid^
Memory CD8^+^ T cell	FS5	TCRβ^mid^CD3^high^CD8^high^CD62L^high^CD44^high^Ly6c^high^CD206^mid^
B cell	FS9	B220^high^CD19^high^CD62L^mid^ CD44^high^Ly6c^low^
	FS12	B220^high^CD19^high^CD62L^low^ CD44^high^Ly6c^low^CD69^high^
	FS17	B220^high^CD19^high^CD62L^low^ CD44^high^Ly6c^low^
	FS18	B220^high^CD19^mid^CD62L^mid^ CD44^high^Ly6c^high^
NK cell	FS6	NK1.1^high^CD11c^low^CD11b^low^Ly6c^high^
NK cell and DC	FS1	NK1.1^high^CD11c^mid^CD11b^mid^Ly6c^low^
Monocyte	FS2	CD11b^high^CD11c^mid^Ly6c^low^F4/80^high^CD206^high^
	FS3	CD11b^high^CD11c^mid^Ly6c^high^F4/80^low^CD206^low^
Granulocyte	FS4	CD11b^high^CD11c^low^Ly6c^high^Gr-1^high^F4/80^low^CD206^low^
Unassigned	FS10	TCRβ^mid^CD3^high^CD4^high^CD8^high^CD62L^high^CD44^high^Ly6c^high^CD206^mid^CD69^high^
	FS13	TCRβ^low^CD3^high^CD8^high^CD62L^low^CD44^high^Ly6c^low^CD69^high^CD206^high^
	FS15	TCRβ^-^CD3^high^CD4^high^CD8^high^CD62L^low^CD44^high^Ly6c^low^CD69^high^CD206^mid^CD25^high^
	FS19	TCRβ^high^CD3^high^CD4^high^CD8^high^CD62L^low^CD44^high^CD206^high^

As shown in **Figure 4C**, in case of the cellular subsets of NK cells, the decrement in the TAA group and the recovery in the drug-treated group, described in the manually gated cellular abundances (see **Figure 3** and **Table S1**), can be attributed to both PhenoGraph clusters (PG#8 and PG#10), as they displayed similar patterns of decrease in the TAA group, followed by recovery in the drug-treated group. Among the nine PhenoGraph clusters assigned as subsets of the B cells, cluster PG#1 exhibited a fivefold increase in cellular abundance in the TAA group [19.4%, delta(TAA-NC) = +15.7%], whereas cluster PG#3 showed a threefold increase in the TAA group [11.5%, delta(TAA-NC) = +8.39%], compared to the NC group. These two PhenoGraph clusters can be considered as the main contributors to the previously mentioned increments in the manually gated B-cell population of the TAA group [delta(TAA-NC) = +21.2%]. In contrast, despite significant recovery being observed in the manually gated populations of the B cells (see **Figure 3** and **Table S1**), cluster PG#1 did not show any significant recovery effect after treatment with 11beta-HSD inhibitor [delta(Drug-TAA) = +0.5%]. Instead, the cellular subsets corresponding to clusters PG#2 and PG#3 appeared to have more dominant roles in the recovery after treatment with 11beta-HSD inhibitor [delta(Drug-TAA) = −4.5% and −7.48% for clusters PG#2 and PG#3, respectively].

Compared to the nine distinctive PhenoGraph clusters shown in **Figure 4A**, the location of the FlowSOM metaclusters assigned as B cells seems indistinctive and similar to the distribution of manually gated B cells. Among the four FlowSOM metaclusters assigned as B cells, metacluster FS#9 appears to be the most important cellular subset, as it displayed the most dominant population, with patterns similar to those observed for the manually gated cellular abundances. In the case of the NK cells, the decrement in the TAA group and the recovery in the drug-treated group, described in manually gated cellular abundances, can be attributed to both FlowSOM metaclusters (FS#1 and FS#6), as both metaclusters were observed as having similar patterns for the manually gated cellular abundances. In cases wherein effector CD4^+^ and memory CD8^+^ T cells showed decrements and recovery patterns similar to those of NK cells, in their manually gated cellular abundances, FlowSOM metacluster FS#20 seems to have a major contribution to the observed pattern for the manually gated cellular abundances of effector CD4^+^ T cells, whereas FlowSOM metacluster FS#5 seems to have a major contribution to the observed pattern for the manually gated cellular abundances of memory CD8^+^ T cells.

### PhenoGraph Clustering of the Manually Gated B Cells and NK Cells

**Figure 5:**
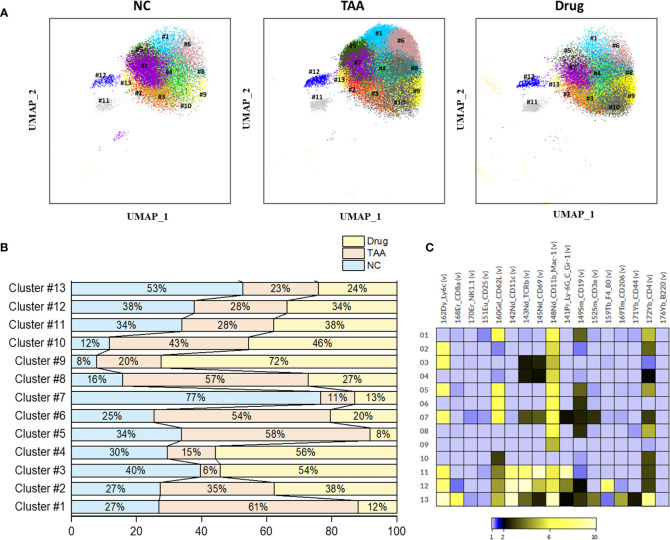
Phenograph sub-clusters of B cells. **(A)** PhenoGraph clusters analysis of manual gated B cells of NC, TAA, and Drug groups. **(B)** Population differences of B cell sub-clusters represented in bar graph (%). **(C)** Intensity of surface marker expression of B cells represented in heat map analysis.

Based on the significant role of B lymphocytes on liver fibrosis, further profiling analysis is necessary to reveal a heterogeneous B-cell subset population. Due to the significant changes observed for the PhenoGraph clusters corresponding to the B cells (PhenoGraph clusters, PG#1, PG#2, PG#3, and PG#5), only the manually gated B cells were isolated and further clustered using the PhenoGraph algorithms based on the intensities of surface markers to precisely identify the B-cell subsets responsible for liver fibrosis. This PhenoGraph clusters of the manually gated B cells are displayed in **Figure 5** and used to further comprehend the nature of drugs and their immunological responses on mPBMC.

PhenoGraph clustering of the manually gated B cells revealed thirteen clusters of B-cell subsets (B-PG#1 to B-PG#13) with the associated surface marker expressions given in **Table 4**. Among these subsets of B cells, the populations of the B-PG #1, #5, #6, and #8 displayed increments in the TAA group followed by the decrements in the Drug-treated group, which seems to be related to the liver fibrosis and drug-induced recovery, respectively. Particularly, the B-PG #1 and #6 clusters correspond well with the previously discovered cluster PG#3 (see **Figures 4A, C**), which are considered as the main contributors of the manually gated B-cell population changes. In contrast, the opposite trends—the decrements in the TAA group followed by the increments in the Drug-treated group—were also observed for the populations of the B-PG clusters #3 and #4. (**Figures 5A–C**).

**Table 4:** Comparison of B cell PhenoGraph clusters with manually gated population and associated marker expression.

Cell assignment	PhenoGraph cluster	Marker expression
B cells	B-PG#1	CD62L ^high^ CD11b_Mac-1^high^ CD19^mid^ CD4^high^
	B-PG#2	Ly6C^high^ CD11b_Mac-1^high^ CD4^mid^
	B-PG#3	Ly6C^high^ TCRβ^low^ CD69^low^ CD11b_Mac-1^high^
	B-PG#4	TCRβ^low^CD69^low^ CD11b_Mac-1^high^ CD4^low^
	B-PG#5	Ly6C^high^ CD62L^high^ CD11b_Mac-1high CD19^mid^ CD4^high^
	B-PG#6	CD62L^high^ CD11b_Mac-1^high^ CD19^mid^
	B-PG#7	Ly6C^high^ CD62L^high^ CD11b_Mac-1^high^ TCRβ^mid^ CD69^mid^ Ly6CGr-1^low^ CD19^low^ CD3e^low^ CD4^low^
	B-PG#8	CD11b_Mac-1^high^ CD19^mid^ CD4^high^
	B-PG#9	CD11b_Mac-1^high^
	B-PG#10	CD62L^mid^ CD4^mid^
	B-PG#11	Ly6C^high^ CD62L^mid^ CD11c^high^ TCRβ^high^ CD69^high^ CD11b_Mac-1^high^ Ly_6G_C_Gr-1^high^
	B-PG#12	Ly6C^high^ CD62L^mid^ CD11c^high^ TCRβ^high^ CD69^high^ CD11b_Mac-1^high^ Ly_6G_C_Gr-1^high^ F4_80^high^
	B-PG#13	Ly6C^high^ CD62L^mid^ CD11c^high^ TCRβ^mid^ CD11b_Mac-1^high^ CD19^low^ CD3e^high^ CD206^high^ CD4^high^

**Figure 6:**
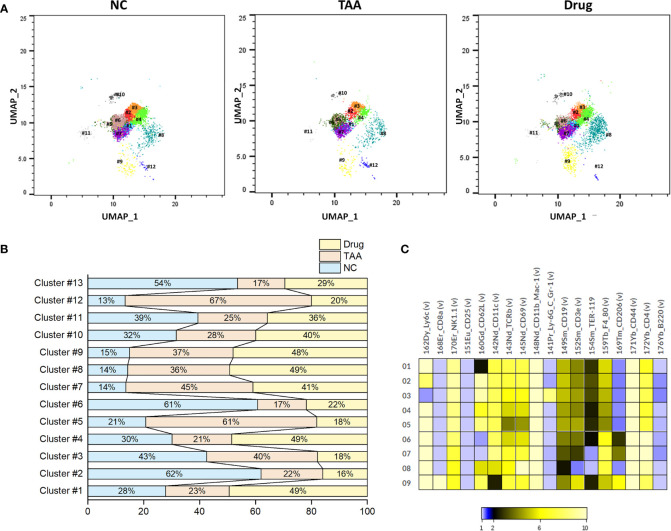
Phenograph sub-clusters of NK cells. **(A)** PhenoGraph cluster analysis of manual gated NK cells of NC, TAA, and Drug groups. **(B)** Population differences of NK cell sub-clusters represented in bar graph (%). **(C)** Intensity of surface marker expression of NK cells represented in heat map analysis.

Similarly, PhenoGraph clustering of the manually gated NK cells revealed nine NK cell subsets, (NK-PG#1 to NK-PG#9) with the associated surface marker expressions given in **Table 5**. Among these NK cell subsets, NK-PG #1 and NK-PG #4 seem related to the recovery of NK cell population followed by the 11beta-HSD inhibitor Drug treatment compared with the TAA group. Likewise, the NK cell population depletion observed in the TAA group compared with the NC group seems related to the clusters NK-PG #2 and NK-PG #6 (**Figures 6A–C**). The NK cell subsets such as NK-PG#5 and NK-PG#7 in the TAA group are responsible for inducing fibrosis through the increment of NK cell sub-clusters (**Figures 6A–C**). In case of T-cell subsets, we could not find any significant differences in population compared with B and NK cells (See **Table S4**, **Supplementary Figure S7**).

**Table 5:** Comparison of NK cell PhenoGraph clusters with manually gated population and associated marker expression.

Cell assignment	PhenoGraph cluster	Marker expression
NK cells	NK-PG#1	Ly6C^high^ NK1^high^ CD11c^high^ TCRβ^high^ CD69^high^ CD11b_Mac-1^high^ CD19^mid^ CD3e^mid^ TER-119^low^ F4_80^high^ CD4^high^ CD44^high^
	NK-PG#2	Ly6C^high^ NK1^high^ CD62L^high^ CD11c^high^ TCRβ^high^ CD69^high^CD11b_Mac-1^high^ CD19^mid^ CD3e^mid^ TER-119^low^ F4_80^high^ CD4^high^ CD44^high^
	NK-PG#3	NK1^high^ CD62L^high^ CD11c^high^ TCRβ^high^ CD69^high^CD11b_Mac-1^high^ Ly_6G_C_Gr-1^high^ CD19^mid^ CD3e^mid^ TER-119^low^ F4_80^high^ CD4^high^ CD44^high^
	NK-PG#4	Ly6C^high^ NK1^high^ CD62L^high^ CD11c^high^ TCRβ^high^ CD69^high^ CD11b_Mac-1^high^ CD19^mid^ CD3e^mid^ TER-119^low^ F4_80^high^ CD4^high^ CD44^high^
	NK-PG#5	Ly6C^high^ NK1^high^ CD62L^high^ CD11c^high^ TCRβ^high^ CD69^high^CD11b_Mac-1^high^ CD19^mid^ CD3e^mid^ TER-119^low^ F4_80^high^ CD4^high^ CD44^high^
	NK-PG#6	Ly6C^high^ NK1^high^ CD11c^high^ TCRβ^high^ CD69^high^CD11b_Mac-1^high^ CD19^mid^ CD3e^mid^ TER-119^low^ F4_80^high^ CD4^high^ CD44^high^ CD206^low^
	NK-PG#7	Ly6C^high^ NK1^high^ CD11c^high^ TCRβ^high^ CD69^high^CD11b_Mac-1^high^ CD19^mid^ CD3e^mid^ F4_80^high^ CD4^high^ CD44^high^ CD206^low^
	NK-PG#8	Ly6C^high^ NK1^high^ CD11c^high^ TCRβ^high^ CD69^high^CD11b_Mac-1^high^ CD19^low^ F4_80^high^ CD4^high^ CD44^high^
	NK-PG#9	Ly6C^high^ CD8a^high^ NK1^high^ TCRβ^high^ CD69^high^CD11b_Mac-1^high^ CD19^low^ F4_80^high^ CD4^high^ CD44^high^ CD19^mid^ CD3e^high^ CD206^high^

## Discussion

Single-cell-based, high-dimensional mass cytometric analysis of mPBMCs collected from three mice groups, namely, NC, TAA, and Drug, was performed to improve our understanding of differences in pathogenesis and to provide insight for the development of reliable noninvasive biomarkers of liver fibrosis. Major immune cell types, as well as changes in their populations, were explored using dimensionality reduction and 2D visualization techniques such as UMAP and MST, combined with a conventional manual gating strategy.

The population of B cells displayed a twofold increase in the TAA group compared to that in the NC group, which recovered slightly following treatment with 11beta-HSD inhibitor. In contrast, the populations of NK cells, effector CD4^+^ T cells, and memory CD8^+^ T cells were significantly reduced in the TAA group compared with those in the NC group, though these decrements recovered slightly in the drug-treated group. These observations are concordant with those of previous studies reporting the accumulation of B cells in NASH livers with inflammation and fibrosis, wherein elevated levels of secreted pro-inflammatory cytokines with antigen-presentation ability were also reported (41). Increase in the B-cell population has also been previously reported to promote fibrosis in a model of acute liver injury (11, 39).

Further identification and quantification of the major immune cell types and their subsets were performed with automated clustering approaches, such as PhenoGraph and FlowSOM, followed by the comparison of their cellular abundances among the three mice groups. The B cell subset corresponding to PhenoGraph cluster PG#3 (CD62L^high^CD44^high^Ly6c^low^ B cell) seems to play a major role in both the development of hepatic fibrosis and recovery after treatment with 11beta-HSD inhibitor. PhenoGraph cluster PG#1 (CD62L^low^CD44^high^Ly6c^low^ B cell) appears to play a dominant role in the development of hepatic fibrosis, whereas the recovery after treatment with 11beta-HSD inhibitor exhibited a higher correlation with PhenoGraph cluster PG#2 (CD62L^high^CD44^high^Ly6c^high^ B cells). These observations suggest that the cellular subsets primarily responsible for the recovery after treatment with 11beta-HSD inhibitor might be different from those responsible for the development of hepatic fibrosis, which is concordant with the observations of high-density spot shifts of the B cells in the UMAP visualization (see **Figure 2A**). Our analysis results also indicated that the CD44 protein of B cells was highly expressed in clusters PG#1 and PG#3 and appears to play an important role in the progression of fibrosis (see **Figure S2**), while the CD62L protein may play an important role during the recovery induced by treatment with 11beta-HSD inhibitor *via* controlling T-cell activation and differentiation. The expression pattern of cell surface glycoprotein CD44 of B-cell population considered as B-regulatory cells helps to maintain the homeostasis of immune response by secreting anti-inflammatory cytokines (42, 43). In mice, the CD62L protein plays an important role to inhibit expansion and progression of steatohepatitis through mediating immune cell infiltration (42). Although further studies involving additional intracellular markers are necessary to confirm our observations, these findings, based on single-cell-based, high-dimensional mass cytometry measurements combined with automated clustering approaches, provide insight into the cellular subsets of B cells during processes associated with hepatic fibrosis and recovery from it.

In case of NK cells, the manually gated NK cell populations displayed progression of hepatic fibrosis through their population depletion and recovery in the Drug-treated group. The NK cells, previously described as comprising ~30% of all liver lymphocytes (44), have been reported to protect the liver against hepatic fibrosis in murine and human models by killing HSC cells (45). The PhenoGraph clusters PG#8 and PG#10, corresponding to the NK cell subsets, displayed similar patterns with that of the manually gated NK cell populations. However, among the PhenoGraph clusters of the manually gated NK cells, NK-PG #1 and NK-PG #4 seem mainly related to the recovery of NK cell population after the 11beta-HSD inhibitor Drug treatment, while NK-PG#5 and NK-PG#7 are mostly related to the induction of fibrosis. The recovery of NK cells may interrupt the accumulation of collagens secreted by myofibroblasts through HSC cell differentiation and is also actively involved in the pathophysiological function to avoid microbial infection in liver (46, 47). The NK cell population depletion observed in the TAA group compared with the NC group seems related to the clusters NK-PG #2 and NK-PG #6 (**Figures 6A–C**).

In summary, based on the mass cytometry exploration of the mPBMCs collected from three mice group, we have observed a significant change in the populations of manually gated B cells, NK cells, effector CD4^+^ T cells, and memory CD8^+^ T cells. Further PhenoGraph clustering analysis revealed that the PhenoGraph cluster PG#1, PG#2, and PG#3 seems to play major roles in the development of hepatic fibrosis and/or recovery after treatment with 11beta-HSD inhibitor. In particular, the CD44, CD62L, and Ly6c proteins of B cells appears to be related to the progression and recovery of fibrosis. Similarly, PhenoGraph clustering analysis of the manually gated NK cells revealed NK-PG #1, #2, #4, #5, #6, and #7, which behave differently from that of NK cells and seems related to the induction and recovery of fibrosis.

Due to the lack of intracellular cytokine markers in the mass cytometry panel used in this study, the findings in this study are not sufficient to propose specific functional roles of each cellular subset. However, although further functional studies involving additional intracellular markers are necessary, the findings in this study, such as the cellular subsets and surface proteins of B cells and NK cells related to the progression of, and/or recovery from, hepatic fibrosis, will provide hints toward elucidating the roles of B cells and NK cells and their subsets related to the pathogenesis of liver fibrosis. Additionally, the results of this pre-clinical study will also help design and interpret the follow-up clinical study using mass cytometry, which will be used for the development of a non-invasive, accurate, and reliable method for the diagnosis of hepatic fibrosis and the efficacy testing of drugs intended for liver fibrosis treatment, as well as to develop reliable noninvasive biomarkers of liver fibrosis.

## Data Availability Statement

The original contributions presented in the study are included in the article/**Supplementary Material**, further inquiries can be directed to the corresponding authors.

## Ethics Statement

The animal study was reviewed and approved by the Institutional Animal Care and Use Committee of Hanyang University. The ethical approval number is 2019-0176A (HY-IACUC-21-0001).

## Author Contributions

Conceptualization, THY and DJ. Validation, SP and HP. Formal analysis, JB, SP, and HP. Investigation, JB, SP, HP, and JK. Writing—original draft preparation, JB and HP. Writing—review and editing, HP, THY, and DJ. Visualization, JB and SP. Supervision, THY and DJ. Funding acquisition, THY and DJ. All authors contributed to the article and approved the submitted version.

## Funding

This research was supported by the Basic Science Research Program through the National Research Foundation of Korea (NRF) (2020R1A6A1A06046728) and Korea Basic Science Institute (National Research Facilities and Equipment Center) (2019R1A6C1030014), both funded by the Ministry of Education.

## Conflict of Interest

THY was employed by Yoon Idea Lab. Co. Ltd.

The remaining authors declare that the research was conducted in the absence of any commercial or financial relationships that could be construed as a potential conflict of interest.

## Publisher’s Note

All claims expressed in this article are solely those of the authors and do not necessarily represent those of their affiliated organizations, or those of the publisher, the editors and the reviewers. Any product that may be evaluated in this article, or claim that may be made by its manufacturer, is not guaranteed or endorsed by the publisher.
