# Supplementary material for: Mass Cytometry Study on Hepatic Fibrosis and Its Drug-Induced Recovery Using Mouse Peripheral Blood Mononuclear Cells
Source: Front Immunol. 2022 Feb 9;13:814030. doi: 10.3389/fimmu.2022.814030 (PMC8863676; doi:10.3389/fimmu.2022.814030)
Supplement: Supplementary file 1 [file DataSheet_1.docx]

Supplementary Material

# Supplementary Figures


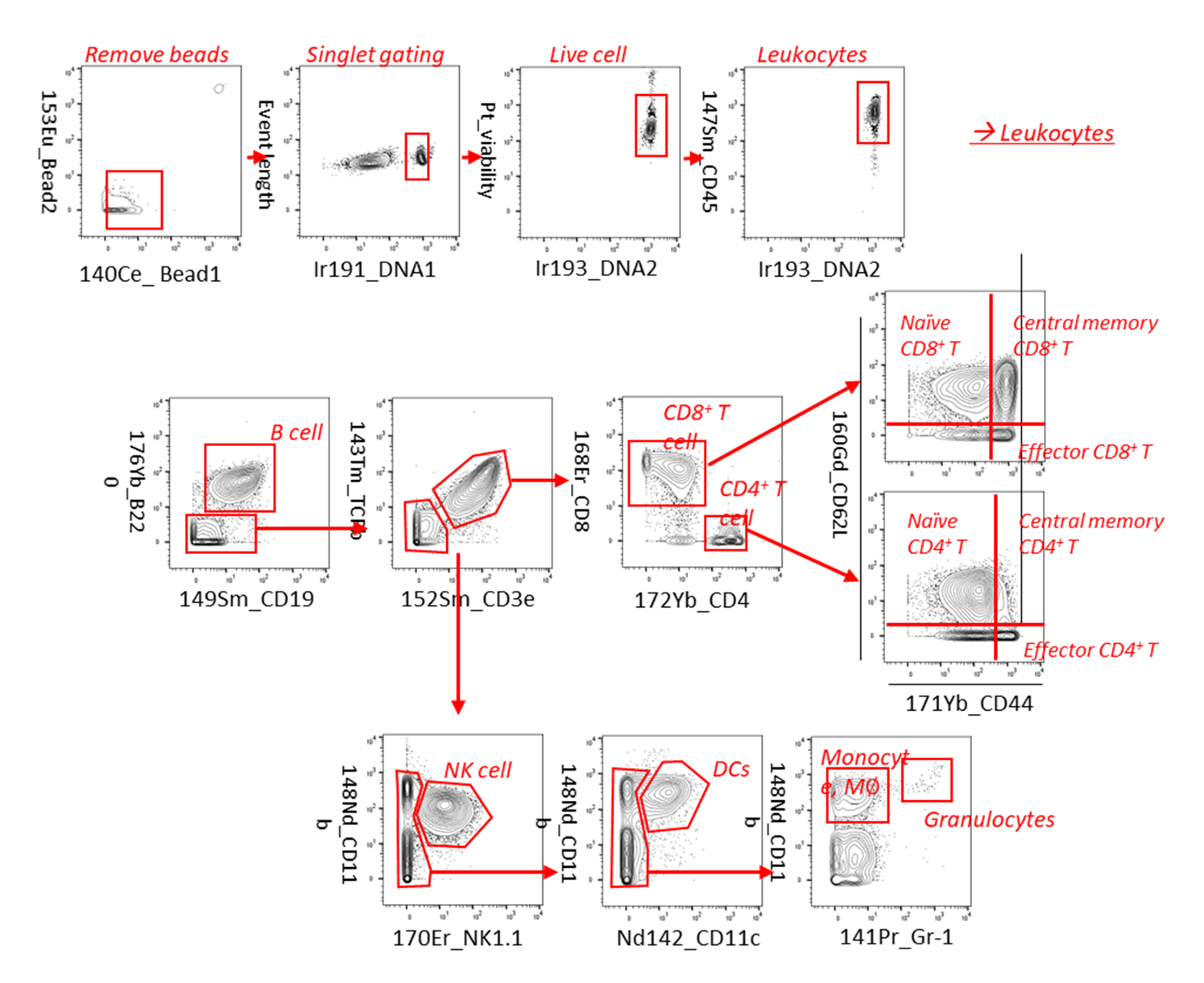


**Figure S1.** Manual gating strategies for identifying the major immune cell types. The gating strategy pipeline and surface markers are used to identify the specific immune cell types respectively.


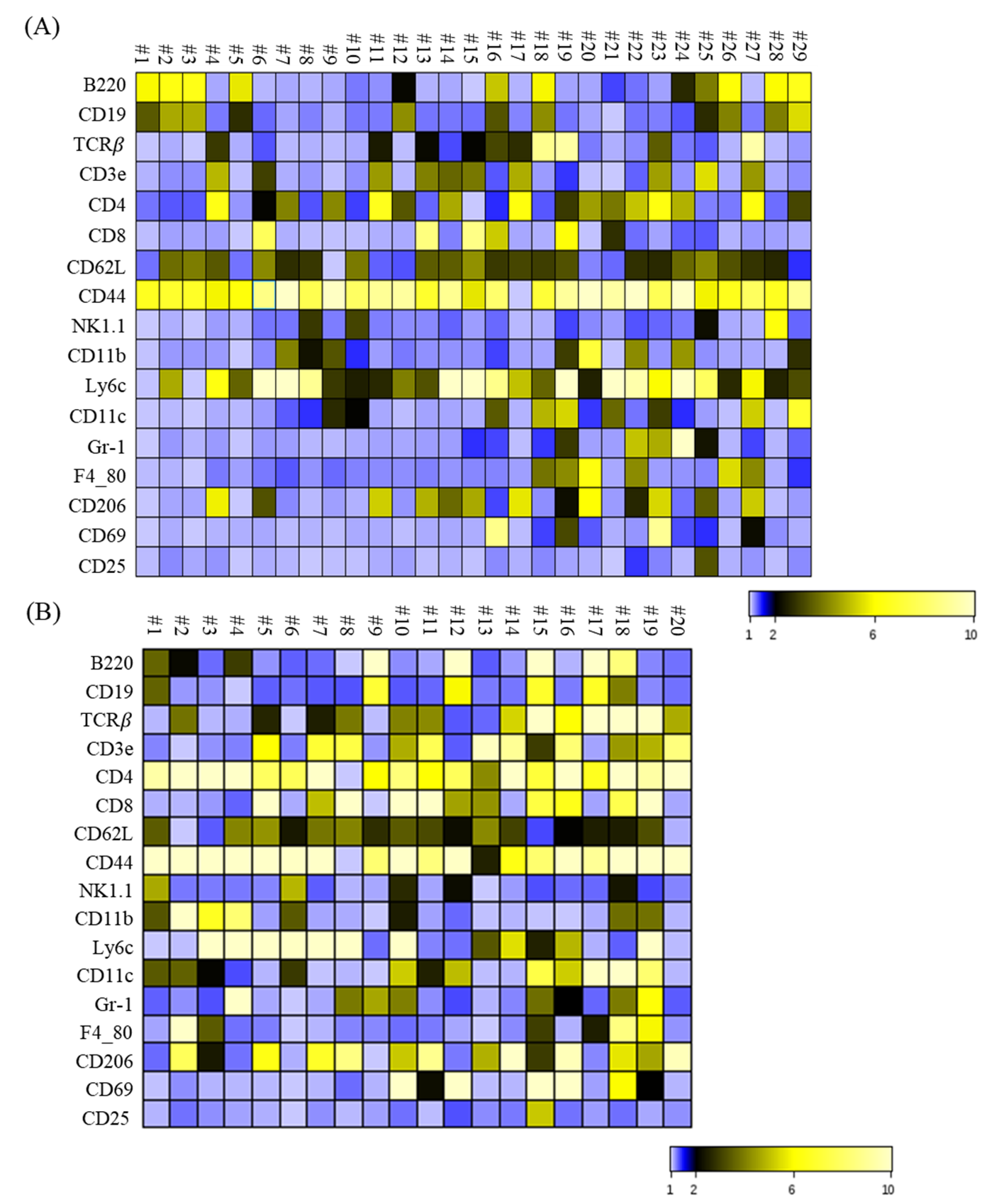


**Figure S2.** Surface Marker expression (A) Intensity of surface markers expression of PhenoGraph clusters of total leukocytes population. (B) Intensity of surface markers expression of flowSOM clusters of total leukocytes population


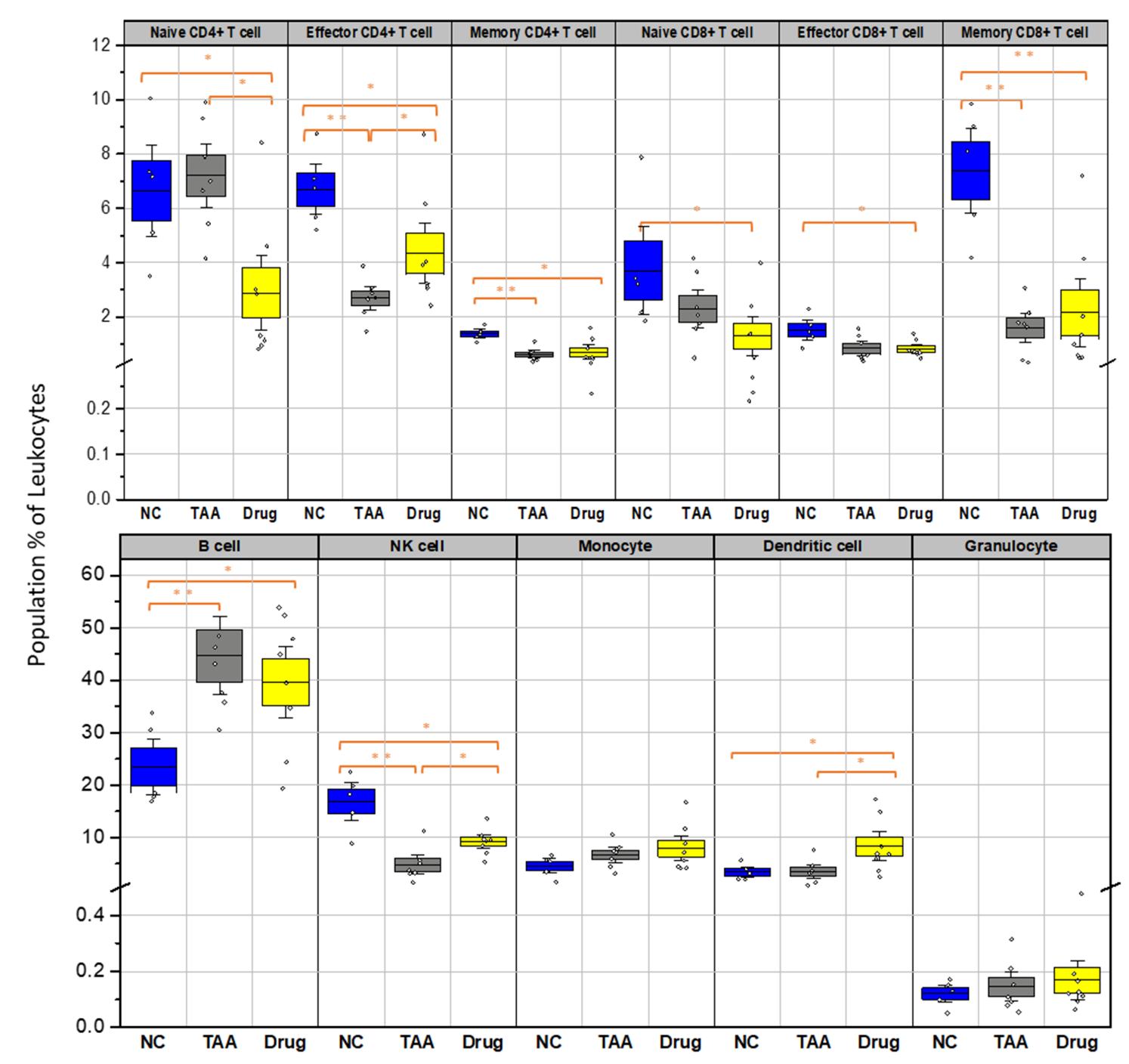


**Figure S3.** The manual gated immune leukocytes population abundance with specific cell types of NC, TAA, and Drug groups. The data represented in boxplots graph and the statistical significant differences considered 0.01 < p < 0.05, 0.001 < p < 0.01, and p < 0.001 have been annotated as *, **, *** respectively.


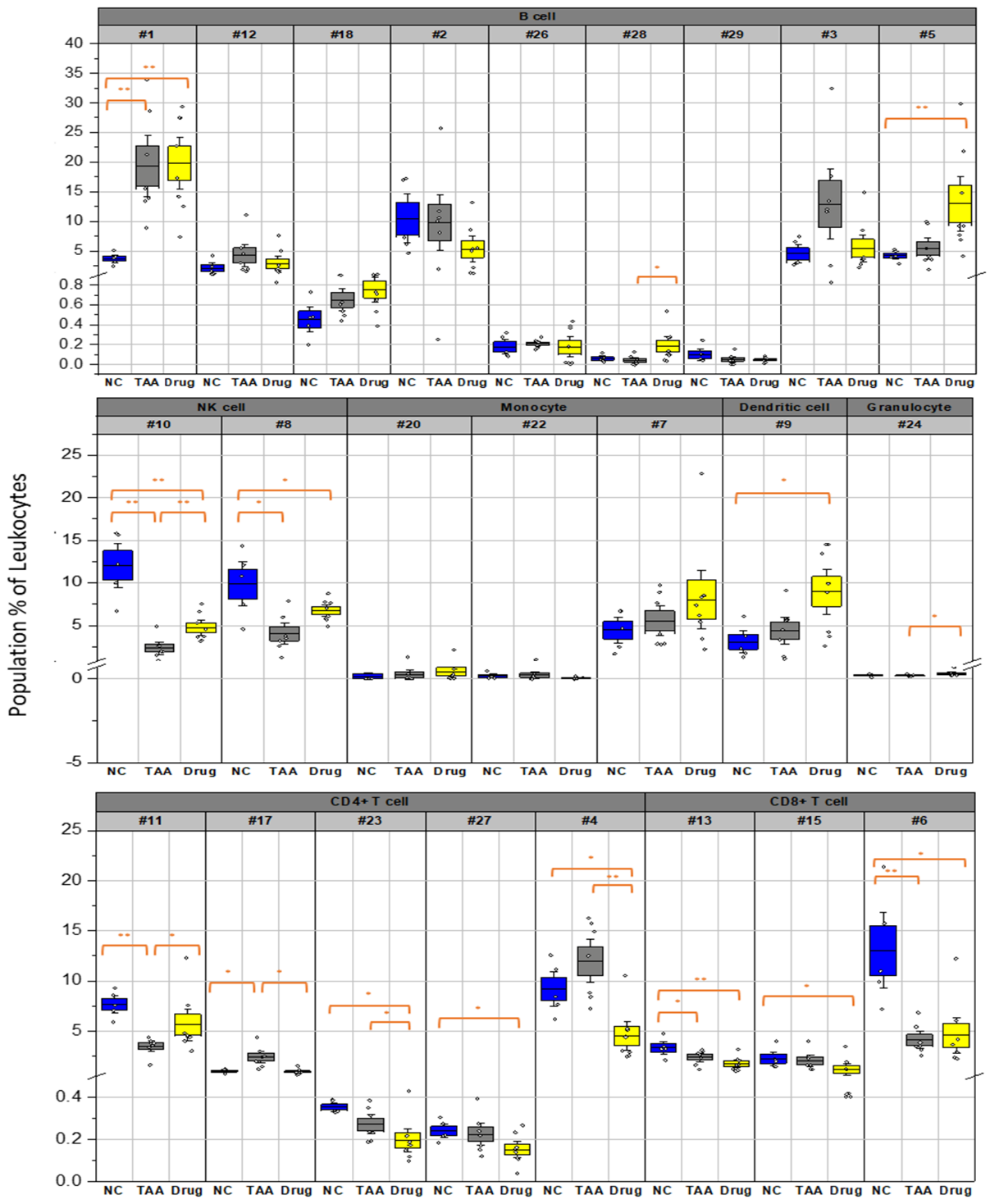


**Figure S4.** The PhenoGraph clustering analysis of manual gated immune leukocytes population abundance with specific clusters of NC, TAA, and Drug groups. The data represented in boxplots graph and the statistical significant differences considered 0.01 < p < 0.05, 0.001 < p < 0.01, and p < 0.001 have been annotated as *, **, *** respectively.


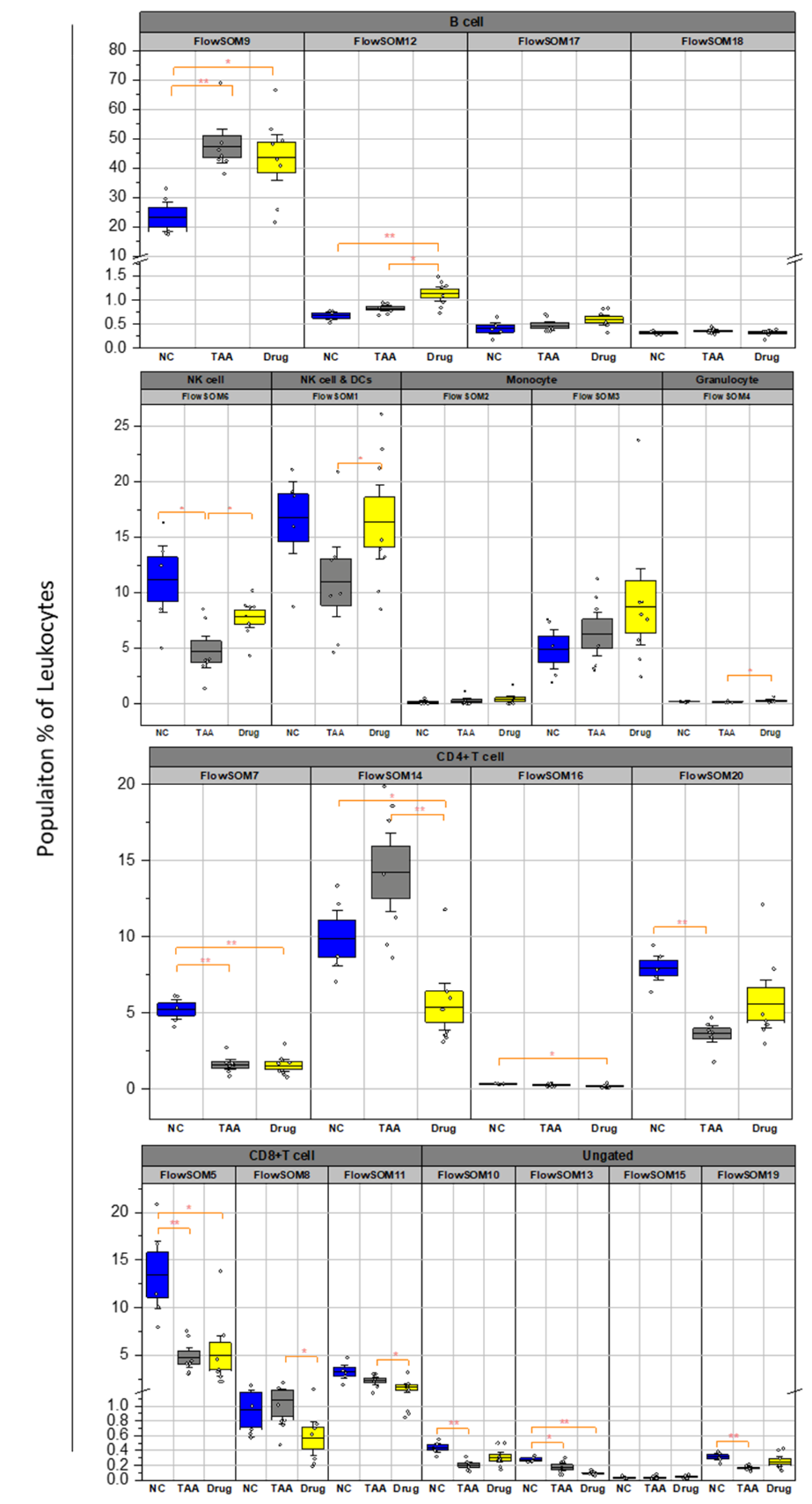


**Figure S5.** The flowSOM clustering analysis of manual gated immune leukocytes population abundance with specific clusters of NC, TAA, and Drug groups. The data represented in boxplots graph and the statistical significant differences considered 0.01 < p < 0.05, 0.001 < p < 0.01, and p < 0.001 have been annotated as *, **, *** respectively.


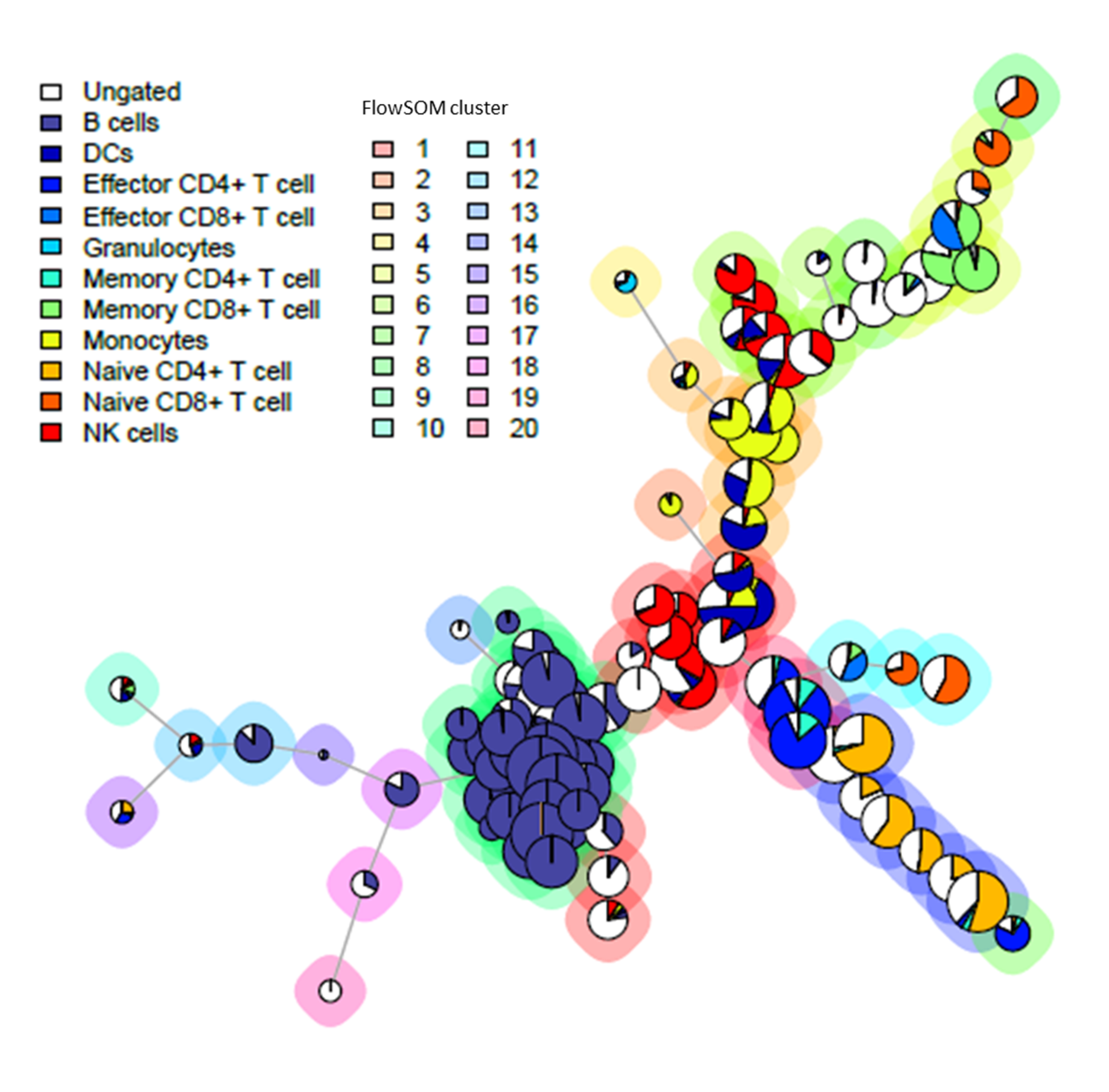


**Figure S6.** FlowSOM minimum spanning tree visualization. The pie chart representation of population abundance aggregated with NC, TAA, and Drug groups

**
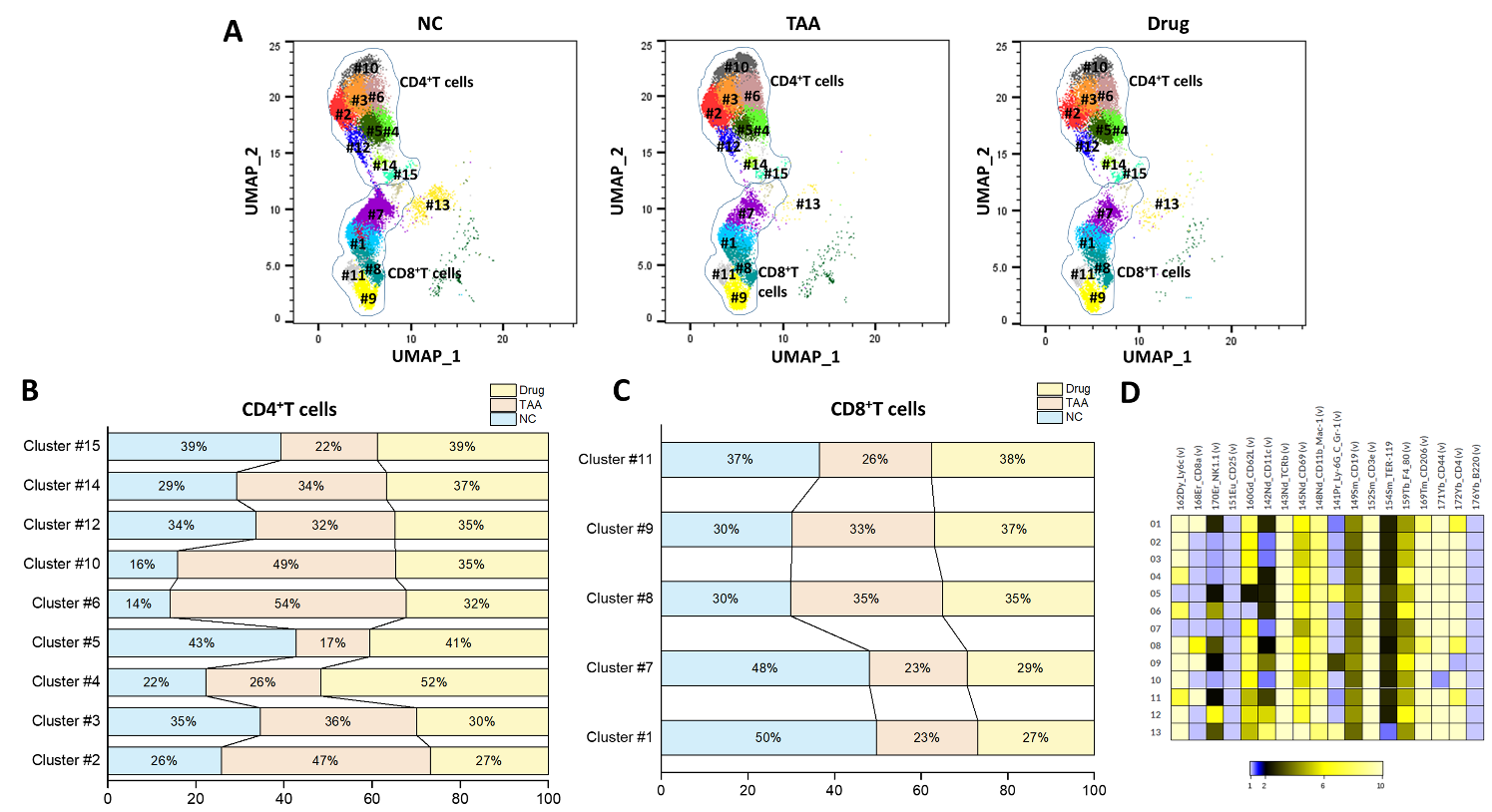
**

**Figure S7.** Phenograph sub-clusters of T cells; (A) PhenoGraph clusters analysis of manual gated CD4^+^T and CD8^+^T cells of NC, TAA, and Drug groups. Population differences of CD4^+^T (B) and CD8^+^T cells (C) sub-clusters represented in bar graph (%). (D) Intensity of surface markers expression of T cells represented in heat map analysis.
