# Supplementary material for: Mass Cytometry Study on Hepatic Fibrosis and Its Drug-Induced Recovery Using Mouse Peripheral Blood Mononuclear Cells
Source: Front Immunol. 2022 Feb 9;13:814030. doi: 10.3389/fimmu.2022.814030 (PMC8863676; doi:10.3389/fimmu.2022.814030)
Supplement: Supplementary file 2 [file DataSheet_2.docx]

Mass cytometry study on hepatic fibrosis and its drug induced recovery using mouse peripheral blood mononuclear cells

Jiwon Bae^1^, Ji Eun Kim^2^ Haribalan Perumalsamy^3^, Sehee Park^1^, Yun Kim^4,5^, Dae Won Jun^2,4,5 ,6^*, Tae-Hyun Yoon^1,3,7,8,^*

^1^Department of Chemistry, College of Natural Sciences, Hanyang University, Seoul 04763, Republic of Korea

^2^Department of Internal Medicine, Hanyang University Hospital, Seoul, 04763, Republic of Korea

^3^Research Institute for Convergence of Basic Science, Hanyang University, Seoul 04763, Republic of Korea

^4^Hanyang Medicine-Engineering-Bio Collaborative & Comprehensive Center for drug development, Hanyang University, Seoul, Korea

^5^Department of Clinical Pharmacology and Therapeutics, Hanyang University Hospital, Seoul, 04763, Republic of Korea

^6^Department of Medical and Digital Engineering, College of Engineering, Hanyang University, Seoul 04763, Republic of Korea

^7^Institute of Next Generation Material Design, Hanyang University, Seoul 04763, Republic of Korea

^8^Yoon Idea Lab. Co. Ltd, Seoul 04763, Republic of Korea

*** Correspondence:**Corresponding Author
[taeyoon@hanyang.ac.kr](mailto:taeyoon@hanyang.ac.kr), [noshin@hanyang.ac.kr](mailto:noshin@hanyang.ac.kr)

**Tables S1**. Cellular abundances of the manually-gated immune cells for the NC, TAA, and Drug mice groups

|  | **NC1*** | **NC2** | **NC4** | **NC5** | **NC6** | **NC7** | **NC8*** | **Average** |
| --- | --- | --- | --- | --- | --- | --- | --- | --- |
| **Granulocytes** | 0.73 | 0.17 | 0.13 | 0.05 | 0.10 | 0.15 | 0.14 | 0.12 |
| **Monocytes** | 15.85 | 5.69 | 5.42 | 3.47 | 1.50 | 6.60 | 4.02 | 4.54 |
| **DCs** | 2.20 | 5.66 | 3.94 | 3.16 | 1.95 | 2.04 | 5.16 | 3.35 |
| **NK cell** | 20.73 | 19.83 | 22.49 | 18.28 | 8.82 | 14.75 | 42.75 | 16.83 |
| **Memory CD4+ T cell** | 0.49 | 1.08 | 1.36 | 1.28 | 1.72 | 1.46 | 1.15 | 1.38 |
| **Effector CD4+ T cell** | 12.20 | 8.74 | 6.74 | 7.08 | 5.67 | 5.21 | 7.46 | 6.69 |
| **Naïve CD4+ T cell** | 0.24 | 7.17 | 5.11 | 3.51 | 10.05 | 7.35 | 0.29 | 6.64 |
| **Memory CD8+ T cell** | 0.49 | 4.19 | 5.76 | 9.01 | 9.84 | 8.10 | 6.31 | 7.38 |
| **Effector CD8+ T cell** | 0.49 | 1.70 | 1.29 | 2.30 | 0.85 | 1.44 | 1.87 | 1.52 |
| **Naïve CD8+ T cell** | 0.49 | 3.21 | 2.18 | 1.85 | 7.87 | 3.43 | 0.72 | 3.71 |
| **B cell** | 1.71 | 17.74 | 18.45 | 16.89 | 33.77 | 30.49 | 7.03 | 23.47 |
| SUM | 55.61 | 75.18 | 72.88 | 66.89 | 82.15 | 81.03 | 76.90 |  |

|  | **TAA1** | **TAA2** | **TAA3** | **TAA4** | **TAA5** | **TAA6** | **TAA7**** | **TAA8** | **Average** | **TAA-NC** |
| --- | --- | --- | --- | --- | --- | --- | --- | --- | --- | --- |
| **Granulocytes** | 0.19 | 0.09 | 0.27 | 0.23 | 0.13 | 0.06 | 0.03 | 0.10 | 0.15 | 0.02 |
| **Monocytes** | 2.89 | 7.22 | 4.31 | 8.05 | 5.79 | 7.01 | 7.65 | 10.51 | 6.54 | 2.12 |
| **DCs** | 1.10 | 4.63 | 1.45 | 4.04 | 3.06 | 3.19 | 13.88 | 7.68 | 3.59 | 0.15 |
| **NK cell** | 5.31 | 3.95 | 1.49 | 11.24 | 3.37 | 3.34 | 9.12 | 5.10 | 4.83 | -12.08 |
| **Memory CD4+ T cell** | 0.69 | 1.01 | 0.66 | 0.45 | 0.53 | 0.32 | 2.08 | 0.66 | 0.62 | -0.76 |
| **Effector CD4+ T cell** | 2.61 | 3.79 | 1.52 | 2.66 | 2.54 | 2.42 | 12.32 | 2.92 | 2.64 | -4.00 |
| **Naïve CD4+ T cell** | 10.47 | 9.22 | 7.96 | 6.91 | 6.83 | 4.15 | 0.58 | 5.40 | 7.28 | 0.56 |
| **Memory CD8+ T cell** | 3.27 | 2.13 | 1.76 | 1.80 | 0.42 | 0.37 | 0.42 | 1.68 | 1.63 | -5.79 |
| **Effector CD8+ T cell** | 1.29 | 1.24 | 0.49 | 0.57 | 0.56 | 0.49 | 2.88 | 0.89 | 0.79 | -0.67 |
| **Naïve CD8+ T cell** | 3.65 | 3.79 | 1.76 | 2.23 | 1.84 | 0.67 | 0.10 | 2.11 | 2.29 | -1.41 |
| **B cell** | 48.14 | 42.68 | 70.76 | 46.28 | 35.68 | 31.20 | 12.57 | 37.62 | 44.62 | 21.24 |
| SUM | 79.62 | 79.75 | 92.43 | 84.45 | 60.77 | 53.22 | 61.61 | 74.67 |  |  |

|  | **Drug1** | **Drug2** | **Drug3** | **Drug4** | **Drug5** | **Drug6** | **Drug7** | **Drug8** | **Average** | **Drug-NC** |
| --- | --- | --- | --- | --- | --- | --- | --- | --- | --- | --- |
| **Granulocytes** | 0.12 | 0.48 | 0.09 | 0.06 | 0.13 | 0.19 | 0.17 | 0.11 | 0.17 | 0.05 |
| **Monocytes** | 5.60 | 11.64 | 16.70 | 4.18 | 4.19 | 7.10 | 8.86 | 4.47 | 7.84 | 3.31 |
| **DCs** | 14.90 | 8.31 | 17.31 | 5.90 | 3.63 | 6.82 | 6.90 | 2.45 | 8.28 | 4.93 |
| **NK cell** | 13.64 | 9.33 | 8.37 | 10.31 | 9.52 | 9.60 | 7.00 | 5.34 | 9.14 | -7.69 |
| **Memory CD4+ T cell** | 0.31 | 0.23 | 1.19 | 0.86 | 0.48 | 1.61 | 0.51 | 0.53 | 0.71 | -0.67 |
| **Effector CD4+ T cell** | 3.07 | 3.22 | 8.72 | 3.92 | 2.42 | 6.16 | 3.13 | 4.03 | 4.33 | -2.36 |
| **Naïve CD4+ T cell** | 0.94 | 1.14 | 0.82 | 3.02 | 4.60 | 8.42 | 2.85 | 1.31 | 2.89 | -3.75 |
| **Memory CD8+ T cell** | 0.51 | 0.49 | 0.99 | 2.02 | 4.15 | 7.19 | 1.35 | 0.59 | 2.16 | -5.22 |
| **Effector CD8+ T cell** | 0.77 | 0.70 | 1.18 | 0.72 | 0.63 | 1.39 | 0.70 | 0.48 | 0.82 | -0.70 |
| **Naïve CD8+ T cell** | 0.24 | 0.52 | 0.22 | 1.37 | 2.40 | 3.98 | 1.38 | 0.27 | 1.30 | -2.41 |
| **B cell** | 39.48 | 34.69 | 19.35 | 53.86 | 47.89 | 24.31 | 44.91 | 52.35 | 39.60 | 16.14 |
| SUM | 79.58 | 70.76 | 74.93 | 86.22 | 80.04 | 76.78 | 77.76 | 71.93 |  |  |

* : Exclude for statistics due to the low number of leukocytes(<1000 cells)

** : Exclude for outlier

**Table S2.** Cellular abundances of the 29 PhenoGraph clusters for the NC, TAA, and Drug mice groups

| **Cluster #** | **Cell type** | **Samples** | | | | | | | |
| --- | --- | --- | --- | --- | --- | --- | --- | --- | --- |
|  |  | NC1* | NC2 | NC4 | NC5 | NC6 | NC7 | NC8* | Average |
| Cluster1 | B cell | 3.41 | 3.16 | 4.03 | 5.08 | 3.76 | 2.41 | 1.58 | 3.69 |
| Cluster2 | B cell | 0.00 | 7.25 | 6.16 | 4.63 | 17.27 | 16.95 | 2.58 | 10.45 |
| Cluster3 | B cell | 0.00 | 3.01 | 2.61 | 3.02 | 7.45 | 6.56 | 1.00 | 4.53 |
| Cluster4 | CD4+ T cell | 2.20 | 11.17 | 7.68 | 6.17 | 12.56 | 8.44 | 0.86 | 9.21 |
| Cluster5 | B cell | 1.22 | 3.99 | 5.26 | 4.98 | 4.07 | 2.83 | 2.01 | 4.23 |
| Cluster6 | CD8+ T cell | 7.56 | 7.20 | 9.94 | 21.40 | 15.73 | 11.00 | 9.18 | 13.05 |
| Cluster7 | monocyte | 1.46 | 6.68 | 4.65 | 2.52 | 1.68 | 6.70 | 4.30 | 4.45 |
| Cluster8 | NK cell | 25.85 | 12.11 | 14.34 | 10.80 | 4.56 | 7.50 | 24.39 | 9.86 |
| Cluster9 | DC | 3.41 | 6.07 | 3.79 | 1.75 | 1.28 | 2.32 | 4.45 | 3.04 |
| Cluster10 | NK cell | 18.54 | 12.20 | 15.60 | 15.83 | 6.67 | 10.00 | 29.12 | 12.06 |
| Cluster11 | CD4+ T cell | 14.63 | 9.27 | 7.57 | 8.57 | 7.07 | 5.91 | 8.75 | 7.68 |
| Cluster12 | B cell | 4.39 | 1.14 | 2.34 | 1.67 | 1.05 | 4.19 | 0.86 | 2.08 |
| Cluster13 | CD8+ T cell | 0.98 | 3.44 | 3.22 | 2.11 | 4.75 | 3.19 | 3.59 | 3.34 |
| Cluster14 | Ungated | 7.80 | 5.01 | 5.43 | 5.29 | 3.17 | 3.14 | 2.01 | 4.41 |
| Cluster15 | CD8+ T cell | 0.73 | 2.19 | 1.52 | 1.45 | 4.01 | 1.97 | 0.43 | 2.23 |
| Cluster16 | Ungated | 1.46 | 1.28 | 1.41 | 1.14 | 1.04 | 1.34 | 1.29 | 1.24 |
| Cluster17 | CD4+ T cell | 0.49 | 1.22 | 0.91 | 0.96 | 1.14 | 0.74 | 0.14 | 0.99 |
| Cluster18 | B cell | 0.24 | 0.39 | 0.48 | 0.20 | 0.48 | 0.73 | 0.14 | 0.45 |
| Cluster19 | ungated | 0.73 | 0.82 | 0.78 | 0.80 | 0.45 | 0.71 | 1.43 | 0.71 |
| Cluster20 | Monocytes | 0.00 | 0.00 | 0.00 | 0.01 | 0.00 | 0.72 | 0.00 | 0.15 |
| Cluster21 | ungated | 1.95 | 0.78 | 0.66 | 0.27 | 0.13 | 0.50 | 0.72 | 0.47 |
| Cluster22 | Monocyte | 0.00 | 0.03 | 0.17 | 0.05 | 0.12 | 0.46 | 0.00 | 0.17 |
| Cluster23 | CD4+ T cell | 0.73 | 0.39 | 0.33 | 0.39 | 0.33 | 0.33 | 0.29 | 0.35 |
| Cluster24 | Granulocyte | 0.98 | 0.24 | 0.27 | 0.07 | 0.23 | 0.23 | 0.14 | 0.21 |
| Cluster25 | ungated | 0.49 | 0.43 | 0.41 | 0.39 | 0.33 | 0.34 | 0.00 | 0.38 |
| Cluster26 | B cell | 0.00 | 0.10 | 0.08 | 0.13 | 0.28 | 0.32 | 0.00 | 0.18 |
| Cluster27 | CD4+ T cell | 0.73 | 0.30 | 0.27 | 0.22 | 0.22 | 0.18 | 0.57 | 0.24 |
| Cluster28 | B cell | 0.00 | 0.04 | 0.02 | 0.06 | 0.12 | 0.06 | 0.00 | 0.06 |
| Cluster29 | B cell | 0.00 | 0.10 | 0.06 | 0.05 | 0.04 | 0.25 | 0.14 | 0.10 |

| **Cluster #** | **Cell type** | **Samples** | | | | | | | | |  |
| --- | --- | --- | --- | --- | --- | --- | --- | --- | --- | --- | --- |
|  |  | TAA1 | TAA2 | TAA3 | TAA4 | TAA5 | TAA6 | TAA7** | TAA8 | Average | TAA-NC |
| Cluster1 | B cell | 21.23 | 15.50 | 8.88 | 13.45 | 28.64 | 33.92 | 11.02 | 13.87 | 19.35 | 15.67 |
| Cluster2 | B cell | 10.06 | 10.58 | 25.76 | 11.74 | 1.95 | 0.25 | 0.80 | 8.08 | 9.78 | -0.68 |
| Cluster3 | B cell | 12.03 | 13.40 | 32.48 | 17.64 | 2.46 | 0.83 | 1.76 | 11.62 | 12.92 | 8.39 |
| Cluster4 | CD4+ T cell | 16.27 | 12.51 | 8.42 | 8.82 | 15.72 | 14.97 | 1.60 | 7.26 | 12.00 | 2.79 |
| Cluster5 | B cell | 5.42 | 3.55 | 1.79 | 3.50 | 9.60 | 9.96 | 1.12 | 4.13 | 5.42 | 1.20 |
| Cluster6 | CD8+ T cell | 5.42 | 6.83 | 2.56 | 3.49 | 3.85 | 3.14 | 2.24 | 3.46 | 4.11 | -8.94 |
| Cluster7 | monocyte | 2.83 | 7.65 | 4.04 | 8.92 | 2.75 | 2.89 | 7.19 | 9.74 | 5.54 | 1.10 |
| Cluster8 | NK cell | 3.77 | 2.60 | 1.21 | 7.89 | 3.17 | 6.00 | 15.50 | 3.50 | 4.02 | -5.84 |
| Cluster9 | DC | 1.10 | 5.72 | 1.38 | 3.34 | 5.66 | 4.48 | 10.38 | 9.10 | 4.40 | 1.35 |
| Cluster10 | NK cell | 2.67 | 1.50 | 0.92 | 4.90 | 1.91 | 2.59 | 5.91 | 1.96 | 2.35 | -9.71 |
| Cluster11 | CD4+ T cell | 3.77 | 4.37 | 1.61 | 3.37 | 3.79 | 4.02 | 16.13 | 3.58 | 3.50 | -4.17 |
| Cluster12 | B cell | 1.57 | 2.95 | 1.91 | 1.90 | 5.52 | 4.54 | 9.11 | 11.11 | 4.21 | 2.14 |
| Cluster13 | CD8+ T cell | 2.67 | 2.99 | 1.17 | 1.64 | 3.10 | 2.81 | 7.03 | 2.34 | 2.39 | -0.95 |
| Cluster14 | Ungated | 1.42 | 2.33 | 0.90 | 1.52 | 1.71 | 1.68 | 2.72 | 1.75 | 1.61 | -2.79 |
| Cluster15 | CD8+ T cell | 4.01 | 2.43 | 1.17 | 1.63 | 2.27 | 1.21 | 0.48 | 1.57 | 2.04 | -0.18 |
| Cluster16 | Ungated | 1.10 | 0.94 | 1.16 | 1.10 | 1.07 | 1.29 | 1.60 | 1.21 | 1.12 | -0.12 |
| Cluster17 | CD4+ T cell | 2.44 | 1.98 | 1.20 | 2.54 | 4.37 | 2.97 | 0.32 | 1.46 | 2.42 | 1.43 |
| Cluster18 | B cell | 0.63 | 0.54 | 0.96 | 0.49 | 0.60 | 0.44 | 0.16 | 0.91 | 0.65 | 0.20 |
| Cluster19 | ungated | 0.24 | 0.28 | 0.20 | 0.46 | 0.32 | 0.39 | 0.32 | 0.47 | 0.34 | -0.38 |
| Cluster20 | Monocytes | 0.00 | 0.02 | 0.02 | 0.00 | 0.06 | 0.30 | 0.48 | 1.28 | 0.24 | 0.09 |
| Cluster21 | ungated | 0.00 | 0.22 | 0.11 | 0.30 | 0.13 | 0.11 | 1.28 | 0.26 | 0.16 | -0.31 |
| Cluster22 | Monocyte | 0.00 | 0.10 | 0.96 | 0.05 | 0.13 | 0.00 | 0.16 | 0.26 | 0.21 | 0.05 |
| Cluster23 | CD4+ T cell | 0.31 | 0.19 | 0.19 | 0.23 | 0.35 | 0.39 | 0.96 | 0.24 | 0.27 | -0.08 |
| Cluster24 | Granulocyte | 0.16 | 0.16 | 0.20 | 0.28 | 0.18 | 0.17 | 0.16 | 0.15 | 0.19 | -0.02 |
| Cluster25 | ungated | 0.16 | 0.19 | 0.16 | 0.35 | 0.22 | 0.14 | 0.32 | 0.24 | 0.21 | -0.17 |
| Cluster26 | B cell | 0.24 | 0.15 | 0.23 | 0.17 | 0.22 | 0.28 | 0.00 | 0.19 | 0.21 | 0.03 |
| Cluster27 | CD4+ T cell | 0.39 | 0.22 | 0.12 | 0.15 | 0.24 | 0.28 | 1.12 | 0.17 | 0.22 | -0.02 |
| Cluster28 | B cell | 0.00 | 0.04 | 0.13 | 0.08 | 0.01 | 0.00 | 0.00 | 0.03 | 0.04 | -0.02 |
| Cluster29 | B cell | 0.08 | 0.05 | 0.16 | 0.04 | 0.00 | 0.00 | 0.16 | 0.05 | 0.05 | -0.05 |

| **Cluster #** | **Cell type** | **Samples** | | | | | | | | |  |
| --- | --- | --- | --- | --- | --- | --- | --- | --- | --- | --- | --- |
|  |  | Drug1 | Drug2 | Drug3 | Drug4 | Drug5 | Drug6 | Drug7 | Drug8 | Average | Drug-NC |
| Cluster1 | B cell | 27.50 | 29.35 | 14.17 | 22.79 | 12.49 | 7.37 | 17.32 | 27.47 | 19.81 | 16.12 |
| Cluster2 | B cell | 2.19 | 1.19 | 1.28 | 8.60 | 13.15 | 5.47 | 5.03 | 5.37 | 5.28 | -5.17 |
| Cluster3 | B cell | 3.90 | 2.76 | 2.17 | 14.93 | 8.53 | 3.60 | 3.97 | 3.64 | 5.44 | 0.91 |
| Cluster4 | CD4+ T cell | 3.05 | 2.56 | 2.46 | 4.42 | 5.10 | 10.56 | 5.25 | 2.89 | 4.54 | -4.67 |
| Cluster5 | B cell | 9.27 | 7.73 | 4.11 | 6.87 | 14.83 | 9.25 | 21.81 | 29.83 | 12.96 | 8.73 |
| Cluster6 | CD8+ T cell | 2.94 | 2.35 | 3.68 | 4.18 | 6.05 | 12.24 | 2.90 | 2.25 | 4.57 | -8.48 |
| Cluster7 | monocyte | 7.37 | 8.52 | 22.87 | 5.42 | 3.42 | 6.22 | 8.30 | 2.18 | 8.04 | 3.59 |
| Cluster8 | NK cell | 8.74 | 4.90 | 5.78 | 7.74 | 7.64 | 7.05 | 6.09 | 5.70 | 6.71 | -3.16 |
| Cluster9 | DC | 14.51 | 14.52 | 13.47 | 4.25 | 3.70 | 9.95 | 8.88 | 2.60 | 8.98 | 5.94 |
| Cluster10 | NK cell | 7.54 | 3.74 | 6.62 | 5.28 | 4.58 | 3.27 | 3.64 | 3.13 | 4.72 | -7.33 |
| Cluster11 | CD4+ T cell | 4.47 | 4.38 | 12.31 | 4.80 | 3.00 | 7.57 | 4.02 | 4.53 | 5.64 | -2.04 |
| Cluster12 | B cell | 1.41 | 7.58 | 1.86 | 0.83 | 5.06 | 1.66 | 2.57 | 2.07 | 2.88 | 0.80 |
| Cluster13 | CD8+ T cell | 1.12 | 1.62 | 1.21 | 1.69 | 2.04 | 3.16 | 2.14 | 1.00 | 1.75 | -1.60 |
| Cluster14 | Ungated | 1.31 | 1.33 | 2.75 | 1.74 | 1.87 | 2.79 | 1.38 | 1.50 | 1.83 | -2.57 |
| Cluster15 | CD8+ T cell | 0.40 | 0.42 | 0.40 | 1.20 | 1.92 | 3.46 | 1.19 | 0.41 | 1.18 | -1.05 |
| Cluster16 | Ungated | 1.36 | 1.54 | 1.27 | 1.98 | 1.80 | 1.46 | 1.43 | 1.62 | 1.56 | 0.32 |
| Cluster17 | CD4+ T cell | 0.72 | 0.86 | 0.68 | 0.92 | 0.95 | 1.54 | 1.14 | 0.69 | 0.94 | -0.05 |
| Cluster18 | B cell | 0.53 | 0.71 | 0.39 | 0.93 | 1.09 | 0.73 | 0.65 | 1.01 | 0.76 | 0.30 |
| Cluster19 | ungated | 0.55 | 0.42 | 0.76 | 0.30 | 0.49 | 0.73 | 0.44 | 0.28 | 0.50 | -0.21 |
| Cluster20 | Monocytes | 0.21 | 2.14 | 0.00 | 0.00 | 0.57 | 0.16 | 0.27 | 0.04 | 0.42 | 0.28 |
| Cluster21 | ungated | 0.21 | 0.34 | 0.97 | 0.26 | 0.14 | 0.17 | 0.10 | 0.12 | 0.29 | -0.18 |
| Cluster22 | Monocyte | 0.00 | 0.02 | 0.03 | 0.00 | 0.15 | 0.06 | 0.02 | 0.03 | 0.04 | -0.13 |
| Cluster23 | CD4+ T cell | 0.10 | 0.12 | 0.19 | 0.22 | 0.17 | 0.43 | 0.19 | 0.15 | 0.19 | -0.16 |
| Cluster24 | Granulocyte | 0.29 | 0.67 | 0.19 | 0.17 | 0.24 | 0.30 | 0.31 | 0.22 | 0.30 | 0.09 |
| Cluster25 | ungated | 0.07 | 0.05 | 0.06 | 0.17 | 0.13 | 0.18 | 0.14 | 0.13 | 0.12 | -0.26 |
| Cluster26 | B cell | 0.02 | 0.01 | 0.00 | 0.02 | 0.37 | 0.18 | 0.39 | 0.44 | 0.18 | 0.00 |
| Cluster27 | CD4+ T cell | 0.04 | 0.10 | 0.23 | 0.15 | 0.16 | 0.27 | 0.14 | 0.11 | 0.15 | -0.09 |
| Cluster28 | B cell | 0.11 | 0.03 | 0.05 | 0.10 | 0.28 | 0.14 | 0.27 | 0.54 | 0.19 | 0.13 |
| Cluster29 | B cell | 0.08 | 0.06 | 0.05 | 0.05 | 0.07 | 0.04 | 0.01 | 0.02 | 0.05 | -0.05 |

* : Exclude for statistics due to the low number of leukocytes(<1000 cells)

** : Exclude for outlier

**Table S3.** Cellular abundances of the 29 FlowSOM clusters for the NC, TAA, and Drug mice groups

| **Cluster #** | **Cell type** | **Samples** | | | | | | | |
| --- | --- | --- | --- | --- | --- | --- | --- | --- | --- |
|  |  | NC1* | NC2 | NC4 | NC5 | NC6 | NC7 | NC8* | Average |
| Cluster1 | NK cell & DCs | 27.80 | 18.71 | 21.12 | 19.09 | 8.76 | 15.96 | 32.71 | 16.73 |
| Cluster2 | Monocyte | 0.00 | 0.01 | 0.01 | 0.02 | 0.02 | 0.51 | 0.00 | 0.11 |
| Cluster3 | Monocyte | 1.22 | 7.40 | 5.21 | 2.58 | 1.91 | 7.60 | 4.16 | 4.94 |
| Cluster4 | Granulocyte | 0.98 | 0.22 | 0.24 | 0.07 | 0.19 | 0.23 | 0.14 | 0.19 |
| Cluster5 | CD8+ T cell | 7.56 | 7.93 | 10.10 | 20.91 | 16.70 | 11.42 | 9.18 | 13.41 |
| Cluster6 | NK cell | 32.20 | 13.73 | 16.34 | 12.46 | 5.05 | 8.54 | 27.55 | 11.22 |
| Cluster7 | CD4+ T cells | 5.12 | 5.37 | 6.07 | 6.15 | 4.55 | 4.08 | 2.44 | 5.24 |
| Cluster8 | CD8+ T cell | 0.00 | 1.00 | 0.57 | 0.63 | 1.85 | 0.68 | 0.00 | 0.95 |
| Cluster9 | B cell | 4.15 | 17.66 | 18.39 | 17.92 | 33.09 | 29.63 | 7.60 | 23.34 |
| Cluster10 | Unassigned | 0.49 | 0.40 | 0.49 | 0.55 | 0.32 | 0.44 | 0.72 | 0.44 |
| Cluster11 | CD8+ T cell | 0.73 | 3.42 | 2.78 | 1.92 | 4.77 | 3.18 | 3.44 | 3.21 |
| Cluster12 | B cell | 1.22 | 0.77 | 0.78 | 0.52 | 0.59 | 0.72 | 0.43 | 0.68 |
| Cluster13 | Unassigned | 0.24 | 0.30 | 0.33 | 0.25 | 0.24 | 0.26 | 0.00 | 0.28 |
| Cluster14 | CD4+ T cells | 2.93 | 12.17 | 8.17 | 7.04 | 13.36 | 8.74 | 0.43 | 9.90 |
| Cluster15 | Unassigned | 0.00 | 0.04 | 0.01 | 0.02 | 0.03 | 0.06 | 0.14 | 0.03 |
| Cluster16 | CD4+ T cells | 0.49 | 0.37 | 0.35 | 0.35 | 0.33 | 0.31 | 0.43 | 0.34 |
| Cluster17 | B cell | 0.24 | 0.39 | 0.47 | 0.18 | 0.35 | 0.65 | 0.29 | 0.41 |
| Cluster18 | B cell | 0.98 | 0.36 | 0.32 | 0.28 | 0.29 | 0.34 | 0.57 | 0.32 |
| Cluster19 | Unassigned | 0.24 | 0.31 | 0.38 | 0.36 | 0.22 | 0.28 | 0.57 | 0.31 |
| Cluster20 | CD4+ T cells | 13.41 | 9.45 | 7.86 | 8.70 | 7.39 | 6.37 | 9.18 | 7.95 |

| **Cluster #** | **Cell type** | **Samples** | | | | | | | | |  |
| --- | --- | --- | --- | --- | --- | --- | --- | --- | --- | --- | --- |
|  |  | TAA1 | TAA2 | TAA3 | TAA4 | TAA5 | TAA6 | TAA7** | TAA8 | Average | TAA-NC |
| Cluster1 | NK cell & DCs | 5.30 | 9.74 | 4.63 | 9.95 | 12.96 | 13.23 | 27.64 | 20.93 | 10.96 | -5.77 |
| Cluster2 | Monocyte | 0.00 | 0.01 | 0.04 | 0.00 | 0.07 | 0.28 | 0.16 | 1.15 | 0.22 | 0.11 |
| Cluster3 | Monocyte | 3.16 | 8.54 | 5.22 | 9.59 | 3.41 | 3.00 | 7.83 | 11.27 | 6.31 | 1.37 |
| Cluster4 | Granulocyte | 0.16 | 0.14 | 0.19 | 0.27 | 0.17 | 0.11 | 0.16 | 0.13 | 0.17 | -0.02 |
| Cluster5 | CD8+ T cell | 7.04 | 7.57 | 3.01 | 4.10 | 4.41 | 3.22 | 1.92 | 4.10 | 4.78 | -8.63 |
| Cluster6 | NK cell | 3.96 | 3.43 | 1.38 | 8.55 | 4.01 | 7.73 | 17.57 | 4.34 | 4.77 | -6.45 |
| Cluster7 | CD4+ T cells | 1.50 | 2.73 | 1.17 | 1.74 | 1.60 | 0.85 | 2.40 | 1.72 | 1.62 | -3.63 |
| Cluster8 | CD8+ T cell | 2.14 | 1.02 | 0.48 | 0.81 | 1.59 | 0.80 | 0.32 | 0.74 | 1.08 | 0.14 |
| Cluster9 | B cell | 48.73 | 42.90 | 68.96 | 46.25 | 42.56 | 44.13 | 13.90 | 38.17 | 47.39 | 24.05 |
| Cluster10 | Unassigned | 0.32 | 0.19 | 0.12 | 0.23 | 0.14 | 0.25 | 0.00 | 0.18 | 0.20 | -0.24 |
| Cluster11 | CD8+ T cell | 2.77 | 3.00 | 1.18 | 1.66 | 3.02 | 2.39 | 6.71 | 2.26 | 2.33 | -0.89 |
| Cluster12 | B cell | 0.71 | 0.69 | 0.94 | 0.77 | 0.83 | 0.91 | 1.44 | 0.93 | 0.83 | 0.15 |
| Cluster13 | Unassigned | 0.08 | 0.12 | 0.08 | 0.24 | 0.25 | 0.30 | 0.16 | 0.17 | 0.18 | -0.10 |
| Cluster14 | CD4+ T cells | 18.59 | 14.13 | 9.49 | 11.27 | 19.88 | 17.63 | 1.76 | 8.65 | 14.23 | 4.34 |
| Cluster15 | Unassigned | 0.00 | 0.02 | 0.03 | 0.02 | 0.08 | 0.06 | 0.16 | 0.03 | 0.03 | 0.00 |
| Cluster16 | CD4+ T cells | 0.40 | 0.18 | 0.18 | 0.22 | 0.34 | 0.36 | 0.80 | 0.24 | 0.27 | -0.07 |
| Cluster17 | B cell | 0.40 | 0.36 | 0.71 | 0.40 | 0.35 | 0.36 | 0.16 | 0.68 | 0.46 | 0.06 |
| Cluster18 | B cell | 0.32 | 0.36 | 0.29 | 0.31 | 0.45 | 0.36 | 0.64 | 0.40 | 0.35 | 0.04 |
| Cluster19 | Unassigned | 0.16 | 0.18 | 0.12 | 0.19 | 0.14 | 0.17 | 0.16 | 0.21 | 0.17 | -0.15 |
| Cluster20 | CD4+ T cells | 4.27 | 4.71 | 1.79 | 3.42 | 3.75 | 3.88 | 16.13 | 3.71 | 3.65 | -4.31 |

| **Cluster #** | **Cell type** | **Samples** | | | | | | | | |  |
| --- | --- | --- | --- | --- | --- | --- | --- | --- | --- | --- | --- |
|  |  | Drug1 | Drug2 | Drug3 | Drug4 | Drug5 | Drug6 | Drug7 | Drug8 | Average | Drug-NC |
| Cluster1 | NK cell & DCs | 22.97 | 26.11 | 21.23 | 10.13 | 13.24 | 13.96 | 14.77 | 8.52 | 16.37 | -0.36 |
| Cluster2 | Monocyte | 0.16 | 1.73 | 0.00 | 0.00 | 0.55 | 0.15 | 0.26 | 0.04 | 0.36 | 0.25 |
| Cluster3 | Monocyte | 8.04 | 9.18 | 23.77 | 5.76 | 4.00 | 7.59 | 9.15 | 2.44 | 8.74 | 3.80 |
| Cluster4 | Granulocyte | 0.28 | 0.65 | 0.19 | 0.13 | 0.18 | 0.29 | 0.27 | 0.19 | 0.27 | 0.08 |
| Cluster5 | CD8+ T cell | 2.71 | 2.25 | 3.53 | 4.53 | 7.07 | 13.83 | 3.22 | 2.22 | 4.92 | -8.49 |
| Cluster6 | NK cell | 10.20 | 6.58 | 8.85 | 8.74 | 8.73 | 7.92 | 7.21 | 6.73 | 8.12 | -3.11 |
| Cluster7 | CD4+ T cells | 0.98 | 0.79 | 1.98 | 1.69 | 1.72 | 2.99 | 1.19 | 1.10 | 1.55 | -3.69 |
| Cluster8 | CD8+ T cell | 0.29 | 0.29 | 0.19 | 0.62 | 0.76 | 1.46 | 0.70 | 0.22 | 0.57 | -0.38 |
| Cluster9 | B cell | 43.02 | 40.78 | 21.61 | 53.24 | 49.36 | 25.70 | 48.19 | 66.59 | 43.56 | 20.22 |
| Cluster10 | Unassigned | 0.28 | 0.14 | 0.49 | 0.27 | 0.32 | 0.50 | 0.27 | 0.18 | 0.31 | -0.13 |
| Cluster11 | CD8+ T cell | 0.93 | 1.35 | 0.90 | 1.65 | 1.96 | 3.16 | 2.02 | 0.85 | 1.60 | -1.61 |
| Cluster12 | B cell | 0.96 | 1.27 | 0.73 | 1.49 | 1.30 | 0.84 | 1.09 | 1.38 | 1.13 | 0.45 |
| Cluster13 | Unassigned | 0.09 | 0.06 | 0.08 | 0.12 | 0.10 | 0.14 | 0.08 | 0.09 | 0.10 | -0.18 |
| Cluster14 | CD4+ T cells | 3.78 | 3.41 | 3.11 | 5.24 | 6.01 | 11.80 | 6.42 | 3.56 | 5.42 | -4.48 |
| Cluster15 | Unassigned | 0.05 | 0.02 | 0.03 | 0.06 | 0.08 | 0.02 | 0.03 | 0.07 | 0.04 | 0.01 |
| Cluster16 | CD4+ T cells | 0.09 | 0.12 | 0.12 | 0.22 | 0.16 | 0.41 | 0.18 | 0.09 | 0.17 | -0.17 |
| Cluster17 | B cell | 0.48 | 0.51 | 0.32 | 0.83 | 0.83 | 0.54 | 0.48 | 0.71 | 0.59 | 0.18 |
| Cluster18 | B cell | 0.28 | 0.37 | 0.34 | 0.18 | 0.32 | 0.39 | 0.33 | 0.36 | 0.32 | 0.00 |
| Cluster19 | Unassigned | 0.17 | 0.13 | 0.43 | 0.20 | 0.30 | 0.41 | 0.20 | 0.17 | 0.25 | -0.06 |
| Cluster20 | CD4+ T cells | 4.23 | 4.26 | 12.11 | 4.91 | 3.01 | 7.89 | 3.93 | 4.50 | 5.61 | -2.35 |

* : Exclude for statistics due to the low number of leukocytes(<1000 cells)

** : Exclude for outlier

**Table S4. Comparison of T cell PhenoGraph clusters with manually gated population and associated marker expression**

| **Cell assignment** | **PhenoGraph Cluster** | **Marker expression** |
| --- | --- | --- |
| T cells | CD4^+^ T-PG#2 | Ly6C^high^ CD62L^high^ TCR𝛽^high^ CD69^high^ CD11b_Mac-1^high^ CD19^mid^ CD3e^high^  F4_80^mid^ CD206^high^ CD4^high^ CD44^high^ |
|  | CD4^+^ T-PG#3 | Ly6C^high^ CD62L^high^ TCR𝛽^high^ CD69^high^ CD11b_Mac-1^high^ CD19^mid^ CD3e^high^  F4_80^mid^ CD206^high^ CD4^high^ CD44^high^ |
|  | CD4^+^ T-PG#4 | Ly6C^high^ CD62L^high^ TCR𝛽^high^ CD69^high^ CD11b_Mac-1^high^ CD19^mid^ CD3e^high^  F4_80^mid^ CD206^high^ CD4^high^ CD44^high^ |
|  | CD4^+^ T-PG#5 | TCR𝛽^high^ CD69^high^ CD11b_Mac-1^high^ Ly_6G_C_Gr-1^high^ CD19^mid^ CD3e^high^  TER-119^low^ F4_80^mid^ CD206^high^ CD4^high^ CD44^high^ |
|  | CD4^+^ T-PG#6 | Ly6C^high^ NK.1^mid^ CD11c^low^ TCR𝛽^high^ CD69^high^ CD11b_Mac-1^high^ CD19^mid^  CD3e^high^ TER-119^low^ F4_80^mid^ CD206^high^ CD4^high^ CD44^high^ |
|  | CD4^+^ T-PG#10 | Ly6C^high^ CD62L^high^ TCR𝛽^high^ CD69^high^ CD11b_Mac-1^high^ CD19^mid^ CD3e^high^  F4_80^mid^ CD206^high^ CD4^high^ |
|  | CD4^+^ T-PG#12 | Ly6C^high^ NK.1^mid^ CD62L^high^ CD11c^low^ TCR𝛽^high^ CD69^high^ CD11b_Mac-1^high^  CD19^mid^ CD3e^high^ TER-119^low^ F4_80^mid^ CD206^high^ CD4^high^ CD44^high^ |
|  | CD4^+^ T-PG#13 | Ly6C^high^ NK.1^mid^ CD62L^high^ CD11c^low^ TCR𝛽^high^ CD69^high^ CD11b_Mac-1^high^  Ly_6G_C_Gr-1^high^ CD19^mid^ CD3e^high^ F4_80^mid^ CD206^high^ CD4^high^ CD44^high^ |
|  | CD8^+^ T-PG#1 | Ly6C^high^ CD8a^high^ CD62L^high^ TCR𝛽^high^ CD69^high^ CD11b_Mac-1^high^ CD19^mid^  CD3e^high^ F4_80^high^ CD206^high^ CD4^high^ CD44^high^ |
|  | CD8^+^ T-PG#7 | CD62L^high^ TCR𝛽^high^ CD69^high^ CD11b_Mac-1^high^ CD19^mid^ CD3e^high^ TER-119^low^ F4_80^mid^ CD206^high^ CD4^high^ CD44^high^ |
|  | CD8^+^ T-PG#8 | Ly6C^high^ CD8a^high^ NK.1^low^ CD62L^high^ TCR𝛽^high^ CD69^high^ CD11b_Mac-1^high^  CD19^mid^ CD3e^high^ F4_80^high^ CD206^high^ CD4^high^ CD44^high^ |
|  | CD8^+^ T-PG#9 | Ly6C^high^ CD8a^high^ CD11c^low^ CD62L^high^ TCR𝛽^high^ CD69^high^ CD11b_Mac-1^high^  Ly_6G_C_Gr-1^low^ CD19^mid^ CD3e^high^ TER-119^low^ F4_80^high^ CD206^high^ CD44^high^ |
|  | CD8^+^ T-PG#11 | Ly6C^high^ CD8a^high^ CD11c^low^ CD62L^high^ TCR𝛽^high^ CD69^high^ CD11b_Mac-1^high^  CD19^mid^ CD3e^high^ TER-119^low^ F4_80^high^ CD206^high^ CD44^high^ |
